# Diagnostic value of olfactory function testing for Alzheimer’s disease and mild cognitive impairment: a systematic review and meta-analysis

**DOI:** 10.3389/fnagi.2025.1551939

**Published:** 2025-05-15

**Authors:** Yuxuan Liu, Yunpeng Cao, Hongquan Wei

**Affiliations:** ^1^Department of Otolaryngology, The First Affiliated Hospital of China Medical University, Shenyang, China; ^2^Department of Neurology, The First Affiliated Hospital of China Medical University, Shenyang, China

**Keywords:** olfactory disorder, olfactory function testing, Alzheimer’s disease, mild cognitive impairment, diagnosis

## Abstract

**Background:**

Alzheimer’s disease (AD) is clinically classified into prodromal (asymptomatic), mild cognitive impairment (MCI) due to AD, and dementia due to AD. This study investigates the diagnostic value of olfactory function testing for AD and MCI.

**Methods:**

Systematic searches of PubMed, Web of Science, Cochrane, and EMBASE databases were conducted up to February 1, 2024. Methodological quality was assessed using the revised Quality Assessment of Diagnostic Accuracy Studies. Effect sizes were combined using a random-effects model (DerSimonian-Laird method), and statistical analyses were conducted using STATA 15.1 and Meta-Disc 1.4 software.

**Results:**

Twenty-five studies with 13,611 participants were included. For diagnosing AD, combined sensitivity (SE) was 0.79 (95% CI: 0.71–0.85), specificity (SP) was 0.78 (95% CI: 0.69–0.84), and AUC was 0.85 (95% CI: 0.82–0.88). For MCI, SE was 0.67 (95% CI: 0.54–0.78), SP was 0.79 (95% CI: 0.71–0.86), and AUC was 0.81 (95% CI: 0.77–0.84). Combined SE and SP for diagnosing AD and MCI were 0.58 (95% CI: 0.46–0.68) and 0.88 (95% CI: 0.78–0.93), with an AUC of 0.78 (95% CI: 0.74–0.82). SE and SP for AD or MCI were 0.83 (95% CI: 0.36–0.98) and 0.94 (95% CI: 0.82–0.98), with an AUC of 0.96 (95% CI: 0.94–0.98).

**Conclusion:**

This systematic review and meta-analysis reveal that olfactory function testing, as a simple, non-invasive, and cost-effective assessment method, demonstrates high diagnostic efficacy in the early identification of AD and MCI, showing promising clinical application.

**Systematic review registration:**

CRD42024520871.

## 1 Introduction

Neurodegenerative diseases, especially Alzheimer’s disease (AD), have emerged as a major global public health concern due to their close association with the aging trend of the population. AD, being the most common type of dementia, comprises approximately 60% of all dementia cases. The incidence of AD increases significantly with age, particularly among individuals aged 65 years old and older (Mayeux and Stern, 2012). Moreover, the incidence of AD is higher in women compared to men (Niu et al., 2017). Mild cognitive impairment (MCI) is generally considered to be the precursor stage of AD, which is a transition from normal to dementia. A longitudinal study over 8 years indicates that participants with MCI are more likely to progress to AD compared to cognitively normal participants [27163817]. Pathologic changes in AD are often found in the MCI stage. AD leads to a decline in thinking, memory, and language skills, personality changes, and certain brain changes, which gradually deteriorate over time. Moreover, the cost of AD care can place a significant financial burden on families. It is irreversible and lacks effective treatment. AD has a high incidence and prevalence worldwide, but the actual prevalence may be underestimated due to underdiagnosis and misdiagnosis (Tahami Monfared et al., 2022). Therefore, early and accurate diagnosis of AD and MCI is essential to achieve effective secondary prevention.

At present, AD and MCI are primarily diagnosed through exclusion. Moreover, these screening tools can assist in diagnosis and risk assessment when used with biomarkers. Common auxiliary diagnostic methods for AD and MCI mainly include clinical assessment (such as Mini-Mental State Examination [MMSE], Montreal Cognitive Assessment, Mini-Cog), brain imaging examination (such as structural and functional imaging, and AD biomarker examination using PET), and laboratory examination such as testing cerebrospinal fluid (CSF), blood, and gene sequence (McKhann et al., 1984; Albert et al., 2011; McKhann et al., 2011; Tian et al., 2021). However, clinical assessment is subject to subjectivity and operator skill, with some degree of diagnostic uncertainty and limited sensitivity (SE) and specificity (SP) for early diagnosis. Brain imaging and laboratory tests are indeed objective. However, these examination methods are not suitable for routine screening due to respective high costs and invasive procedures (Zou et al., 2016). Hence, finding an economical and accurate diagnostic method is of great significance. Olfactory testing is widely used in otorhinolaryngology departments. In recent years, a significant correlation has been observed between olfactory impairment and neurodegenerative diseases. Olfactory decline has been found to be consistent with the neurodegenerative process in AD patients (Son et al., 2021). It has been mentioned that pathological indicators related to olfactory impairment may serve as biomarkers for early detection of AD (Winchester and Martyn, 2020; Fatuzzo et al., 2023). Moreover, olfactory testing is effective in diagnosing patients with AD and MCI (Audronyte et al., 2023a). Olfactory impairment in AD and MCI may be closely related to early pathological changes in these diseases.

Regarding pathological changes in the olfactory bulb and olfactory cortex, early pathological changes in AD typically first appear in the two regions. These regions are primary processing centers for olfactory signals. Early deposition of amyloid plaques and neurofibrillary tangles in these regions can significantly affect olfactory signal transmission and processing (Lachen-Montes et al., 2019). One study indicated that in the brains of AD patients, the neuron density of the olfactory bulb was significantly reduced, and the structure of the olfactory cortex was also degraded. The olfactory bulb of patients with AD showed obvious atrophy by high-resolution nuclear magnetic resonance imaging (MRI) (Thomann et al., 2009). These changes directly lead to a decrease in olfactory function. The degeneration of neurons in the olfactory bulb and the appearance of neurofibrillary tangles in the olfactory cortex disrupt the pathway of olfactory signal transmission, leading to distortion and loss of olfactory signals during transmission (Xing et al., 2023). In addition, compared to younger adults, older adults have reduced activation of central olfactory regions during olfactory processing, including the internal olfactory cortex and hippocampus. Collectively, these data support the use of olfactory assessment for early detection of AD (Wilson et al., 2007).

Regarding the roles of tau protein and amyloid protein, the pathological changes of the two proteins are symbolic pathological features of AD. Furthermore, these pathological proteins are preferentially accumulated in olfactory structures. Tau protein mainly exists in neurons and can bind and stabilize microtubules. In addition, it also regulates axonal transport, synaptic function, and signal pathway (Ubeda-Bañon et al., 2020). The formation of amyloid plaques and neurofibrillary tangles caused by excessive phosphorylation of tau protein can also be observed in the olfactory pathway (Franco et al., 2024). The accumulation of these pathological proteins causes neuronal dysfunction and death, which disrupts olfactory signaling and processing. It has been shown that pathological deposition due to an imbalance in amyloid production and clearance induces tau protein aggregation through unknown mechanisms (Masters et al., 2015).

Olfactory impairment may reflect early neuropathological changes in AD and MCI, providing a new perspective for early intervention (Marin et al., 2018). It also emphasizes the potential value of olfactory function assessment in the diagnosis of neurodegenerative diseases. Several previous studies have explored the performance of olfactory testing in identifying cognitive impairment (Kjelvik et al., 2007; Conti et al., 2013; Zhao et al., 2020; Audronyte et al., 2023b). However, current research has not yet provided a quantitative summary of this issue, making it difficult to systematically evaluate the scientific basis and diagnostic significance of using olfactory testing for diagnosing AD or MCI. Although there is evidence of a link between olfactory decline and AD, a quantitative analysis of the data has not been carried out (Sun et al., 2012). A study has confirmed the correlation between olfactory detection and AD. However, it has not established a relationship with the diagnosis of MCI (Silva et al., 2018). In contrast, Adam et al. did not address the correlation between olfactory testing and diagnosis (Bothwell et al., 2023). Without a comprehensive quantitative analysis, it is challenging to accurately measure the sensitivity, specificity, and correlation of olfactory testing with pathological states, thereby limiting their application in clinical diagnostics. Through the integrated analysis of extensive data, the accuracy of olfactory testing in the early detection of AD and MCI can be more noticeably validated, providing more valuable diagnostic tools for clinical use, especially in the context of early diagnosis and intervention. Given the urgent need for early diagnosis of AD and MCI, this study aimed to carry out a meta-analysis to quantitatively summarize diagnostic and association data between olfactory disorders and these two diseases in the existing literature. Through this analysis, we hope to determine the value of olfactory function as a potential biomarker for the prediction or diagnosis of AD and MCI. This could offer enhanced opportunities for early intervention and treatment, thereby alleviating the burden of disease on patients and their families.

## 2 Methods

This study was performed in the framework of the Preferred Reporting Items for Systematic Review and Meta-Analysis of Diagnostic Test Accuracy Studies (McInnes et al., 2018) and Meta-analysis of Observational Studies in Epidemiology (Stroup et al., 2000) guidelines. The protocol of the current study was registered for meta-analysis in the International Prospective Register of Systematic Reviews (registration No. CRD42024520871).

### 2.1 Retrieval strategy

Literature searches were performed in PubMed, Embase, Cochrane Library and Web of Science databases with a limited timeframe of database build time to February 1, 2024. The search was performed with a combination of subject terms and free words, including: (Alzheimer Disease OR dementia OR mild Cognitive Dysfunction OR mild cognitive disability) AND (Olfaction Disorders OR impaired olfaction OR nasal Proteins OR nasal marker) AND (Diagnosis OR Prognosis). The specific search strategy used is detailed in Supplementary File S1.

### 2.2 Literature screening

Inclusion criteria: (i) Study participants: healthy adults, AD patients, MCI patients, or MCI due to AD patients; (ii) Diagnostic methods to be evaluated: olfactory function testing, such as Sniffin’ Sticks odor identification test (SS-OIT), threshold, discrimination, and identification score, University of Pennsylvania smell identification test (UPSIT), brief smell identification test (B-SIT), pocket smell test, and Sniffin’ Sniffing screen test; (iii) Outcome indicator: Patients had to meet clear diagnostic criteria such as the DSM (Diagnostic and Statistical Manual of Mental Disorders) or NIA-AA (National Institute on Aging-Alzheimer’s Association) guidelines. Assessment tools included in the study included the Mini-Mental State Examination (MMSE), Clinical Dementia Rating Scale (CDR), and Alzheimer’s Disease Rating Scale-Cognitive Component (ADAS-Cog). A comprehensive evaluation was performed with the use of cerebrospinal fluid biomarker testing (Aβ42, tau), imaging studies (head CT or MRI), and blood tests; (iv) Reported diagnostic accuracy outcomes or correlation data. In the analysis of diagnostic accuracy, sufficient data need to be available or can be derived from raw data, such as SE, SP, true positive (TP), false positive (FP), true negative (TN), and false negative (FN). Correlational data are required to report the relative risk, odds ratio (OR), risk ratio, and their 95% confidence intervals (CIs) related to the outcome, or raw data that can be used for calculation.

Exclusion criteria: (i) reviews, meta-analyses, conference abstracts, editorials, letters, replies, case reports, commentaries, short surveys, and notes; (ii) clinical trials, animal or *in vitro* studies; (iii) duplicates and unavailability of full text; (iv) unavailability of extraction of outcome metrics; and (v) non-English language literature.

Two reviewers, YXL and HQW, independently screened the literature based on the aforementioned criteria by first importing the retrieved literature into Endnote 20 and removing duplicates. After removing duplicates, the titles and abstracts were read, and the preliminary eligible studies were screened. Subsequently, the full text was downloaded, and further full-text reading was performed to screen the studies for meta-analysis. Any discrepancies encountered during the screening process of the study were resolved through discussion between the reviewers.

### 2.3 Data extraction and quality assessment

Two reviewers, HQW and YXL, independently extracted data from the final included studies. The extracted information included first author, publication year, country, basic information of study participants (sample size, age of included participants), test methods, test indicators, cutoff values, SP, SE, TP, FP, FN, and TN. These metrics were either directly provided in the original data or can be calculated from the available data sources. During the study screening process, any divergent opinions encountered were resolved through discussions among the reviewers.

Diagnostic studies included were evaluated for quality and applicability by two independent reviewers (HQW and YXL) with the use of the Quality Assessment of Diagnostic Accuracy Studies-2 (QUADAS-2) tool (Whiting et al., 2011). There are four risk of bias domains (patient selection, index test, reference standard, and flow and timing) and three applicability domains (patient selection, index test, and reference standard) in QUADAS-2. The risk of bias can be considered low if all key questions were answered in the affirmative. Conversely, a high risk of bias was indicated if any of the informational inquiries were answered negatively. When there was insufficient information available to make a definitive judgment, it was categorized as an unclear risk of bias. The two reviewers worked together to discuss the differences and reach a consensus. Rev. Man 5.4 software was used to fill in and graph the quality of the included studies.

Correlational studies included were assessed for quality using the Newcastle-Ottawa scale (NOS) or according to the methodology checklist of the Agency for Healthcare Research and Quality. NOS was conducted to assess quality based on eight questions, primarily grouped into three domains. The maximum score achievable was 2 for comparability and 1 for each of the remaining seven criteria. A study scoring between 7 and 9 points was considered high quality, while a score ranging from 4 to 7 points was categorized as moderate quality. The Joanna Briggs Institute Quality Assessment Scale (JBI) was used to assess the risk of bias in cross-sectional studies. The tool used eight criteria to evaluate the overall methodological quality of the included studies, including sample inclusion criteria, description of the topic and setting, valid and reliable exposure measurements, objective and standardized status measurements, identification of confounders, strategies for coping with confounders, valid and reliable measurement results, and appropriate statistical analyses. All items in these tools had four options: yes (1 point), no (0 points), unclear (0 points), or not applicable (0 points). The included studies were classified into high quality (80% and above), moderate quality (60–80%), and low quality (<60%) based on the entries in the evaluation tool mentioned above. The assessment was completed and cross-checked by two researchers, and in case of disagreement, the final conclusions were obtained through discussion.

### 2.4 Data integration and statistical analysis

The primary outcome measure was the diagnostic ability of olfactory detection for AD disease and MCI, while the secondary outcome measure was the magnitude of the association between olfactory function and AD disease and MCI.

Heterogeneity was statistically quantified using Cochran’s Q-test and Higgins *I*^2^. If *p* < 0.1 or *I*^2^ > 50%, it signified significant heterogeneity, in which case a random-effects model was employed. Otherwise, a fixed-effects model was utilized. If heterogeneity was high, subgroup and meta-regression analyses were employed to explore sources of heterogeneity. For diagnostic accuracy analysis, we performed a meta-analysis using Stata software (version 15.0; Stata Corporation, TX, USA) and Meta-Disc 1.4. The analysis was performed by the MIDAS module in the bivariate mixed-effects model. The combined values of SE, SP, positive likelihood ratio (PLR), negative likelihood ratio (NLR), diagnostic score (DS), and diagnostic odds ratio (DOR) were computed and visualized using forest plots. Greater values of DS and DOR signified superior diagnostic performance. By plotting the summary receiver operating characteristic (SROC) curve, the area under the SROC curve (AUC) was obtained. When the AUC values were between 0.5 and 0.7, 0.7–0.9, and 0.9–1.0, the diagnostic performance was considered low, moderate, and high, respectively. Spearman’s correlation coefficient and its associated *p*-value were employed to detect the presence of a threshold effect. If *p* > 0.05, it suggested no heterogeneity among studies due to a threshold effect. Meta-analysis was conducted by the mean of Stata 15.0 in the correlation analysis. A *p* < 0.05 was used to demonstrate that the combined statistics of the included studies were statistically significant. A Deek’s funnel plot was drawn to assess for publication bias among diagnostic outcome studies, where *p* > 0.05 indicated no significant publication bias. For correlation analysis, a funnel plot was generated to assess the presence of publication bias in the included studies. Moreover, Egger’s or Begg’s methods were employed for statistical testing. For outcomes with significant publication bias, the impacts of publication bias on results were measured by the trim and fill method.

## 3 Results

### 3.1 Literature screening results and flowchart

**Figure 1:**
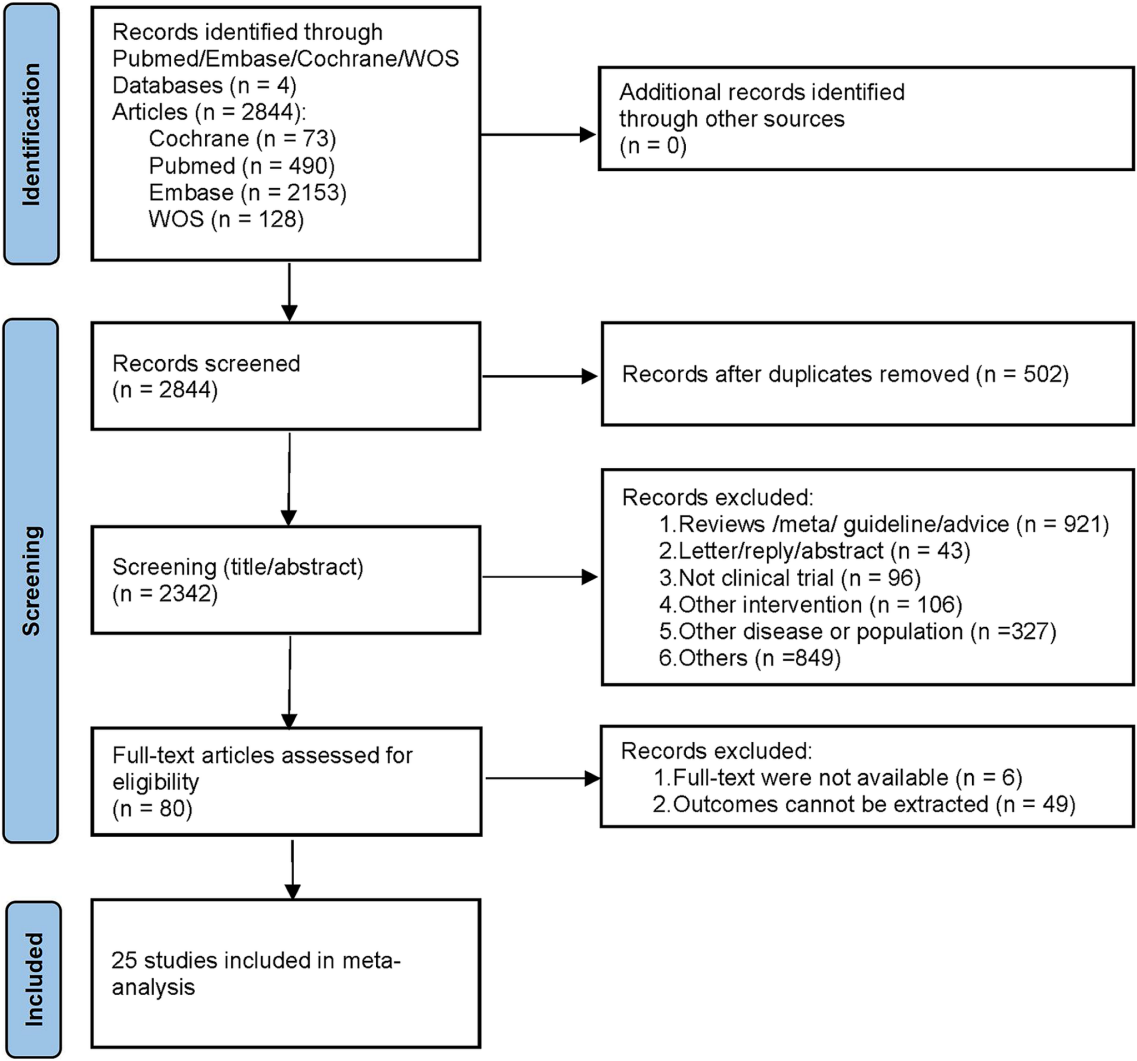
The literature screening process.

A total of 2,844 articles were retrieved initially from the database search, and no additional studies were found through reference scanning. After removing duplicates, 2,342 articles were screened based on their titles and abstracts. Among these, 2,262 articles were excluded for not meeting the inclusion criteria, leaving 80 articles for detailed full-text review. The rescreening process excluded articles with incompatible study objectives, incompatible outcome data, and unavailable full text. Ultimately, a total of 25 studies were included in this meta-analysis (Devanand et al., 2000; Suzuki et al., 2004; Eibenstein et al., 2005; Kjelvik et al., 2007; Djordjevic et al., 2008; Fusetti et al., 2010; Jimbo et al., 2011; Makowska et al., 2011; Conti et al., 2013; Velayudhan et al., 2015; Liang et al., 2016; Moon et al., 2016; Quarmley et al., 2017; Tonacci et al., 2017; Woodward et al., 2017; Yu et al., 2018; Churnin et al., 2019; Kim et al., 2019; Pellkofer et al., 2019; Zhao et al., 2020; Dong et al., 2022; Fukumoto et al., 2022; Pusswald et al., 2022; Audronyte et al., 2023b; Mi et al., 2023). The literature screening process is shown in Figure 1.

### 3.2 Basic characteristics of the included studies

The 25 included studies were from 12 countries (5 from China, 1 from Lithuania, 4 from Italy, 4 from the United States, 2 from South Korea, 1 from the United Kingdom, 3 from Japan, 1 from Norway, 1 from Poland, 1 from Austria, 1 from Germany, and 1 from Canada), involving a total of 13,611 patients, with 3,881 males and 5,023 females. In addition, there were six studies without gender-specific information. The age range of participants in the included studies was 42–91 years old. Supplementary File S2 provides a detailed overview of the basic characteristics of the included studies.

### 3.3 Quality assessment

The QUADAS-2 standard was used for quality assessment as shown in Supplementary Figure S1. There were four criteria in QUADAS-2 for evaluating most included studies: patient selection, index test, reference standard, and flow and timing. Due to being case–control studies, 24 studies were deemed to have a high risk of bias in patient selection. In the context of the index test, nine articles were classified as having a high risk of bias because the threshold was not pre-set, while 10 articles had an unclear risk due to insufficient information provided. In the context of the reference standard, 15 articles were assessed to have an unclear risk due to insufficient information provided. In the field of flow and timing, five articles had a high risk of bias due to unreasonable testing time intervals, and three articles had an uncertain risk due to insufficient information. Because this meta-analysis included numerous case–control studies, the primary risk of bias among the included studies was related to patient selection. In addition, Tables 1, 2 respectively display the detailed quality assessments conducted for each study in accordance with the guidelines of NOS and JBI.

**Table 1:** Detailed quality assessments conducted for each study in accordance with the guidelines of NOS.

Study	Selection				Comparability	Outcome			Quality scores
	Representativeness of the exposed cohort	Selection of the nonexposed cohort	Ascertainment of exposure	Demonstration that outcome of interest was not present at start of study	Comparability of cohorts on the basis of the design or analysis^&^	Assessment of outcome	Was follow-up long enough for outcomes to occur^#^	Adequacy of follow-up of cohorts^¶^	
Zhao et al. (2020)	*	*	*	*	**	*	*	*	9
Pusswald et al. (2022)	*	*	*	*	–	*	*	*	7

& We selected “age and sex” as the most important adjusting factors.

# A mean duration of follow-up of at least 2 years was considered as long enough for outcome to occur in this meta-analysis.

¶ It was regarded adequate when the follow-up rate was at least 80%.

**Table 2:** The detailed quality assessments conducted for each study in accordance with the guidelines of JBI.

Study	Q1	Q2	Q3	Q4	Q5	Q6	Q7	Q8	Quality
Mi et al. (2023)	Yes	Yes	Yes	Yes	Yes	Yes	Yes	Yes	High
Jimbo et al. (2011)	Yes	No	Yes	Yes	No	No	Yes	Yes	Moderate
Churnin et al. (2019)	Yes	Yes	Yes	Yes	Yes	Yes	Yes	Yes	High
Liang et al. (2016)	Yes	Yes	Yes	Yes	Yes	Yes	Yes	Yes	High

The JBI Checklist provides quality criteria for prevalence studies in eight distinct items. The table indicates which items have been fulfilled for each included study, respectively:

Q1: Were the criteria for inclusion in the sample clearly defined?

Q2: Were the study subjects and the setting described in detail?

Q3: Was the exposure measured in a valid and reliable way?

Q4: Were objective, standard criteria used for measurement of the condition?

Q5: Were confounding factors identified?

Q6: Were strategies to deal with confounding factors stated?

Q7: Were the outcomes measured in a valid and reliable way?

Q8: Was appropriate statistical analysis used?

### 3.4 Meta-analysis results

#### 3.4.1 Efficiency analysis of olfactory function testing for diagnosing healthy people and AD

**Figure 2:**
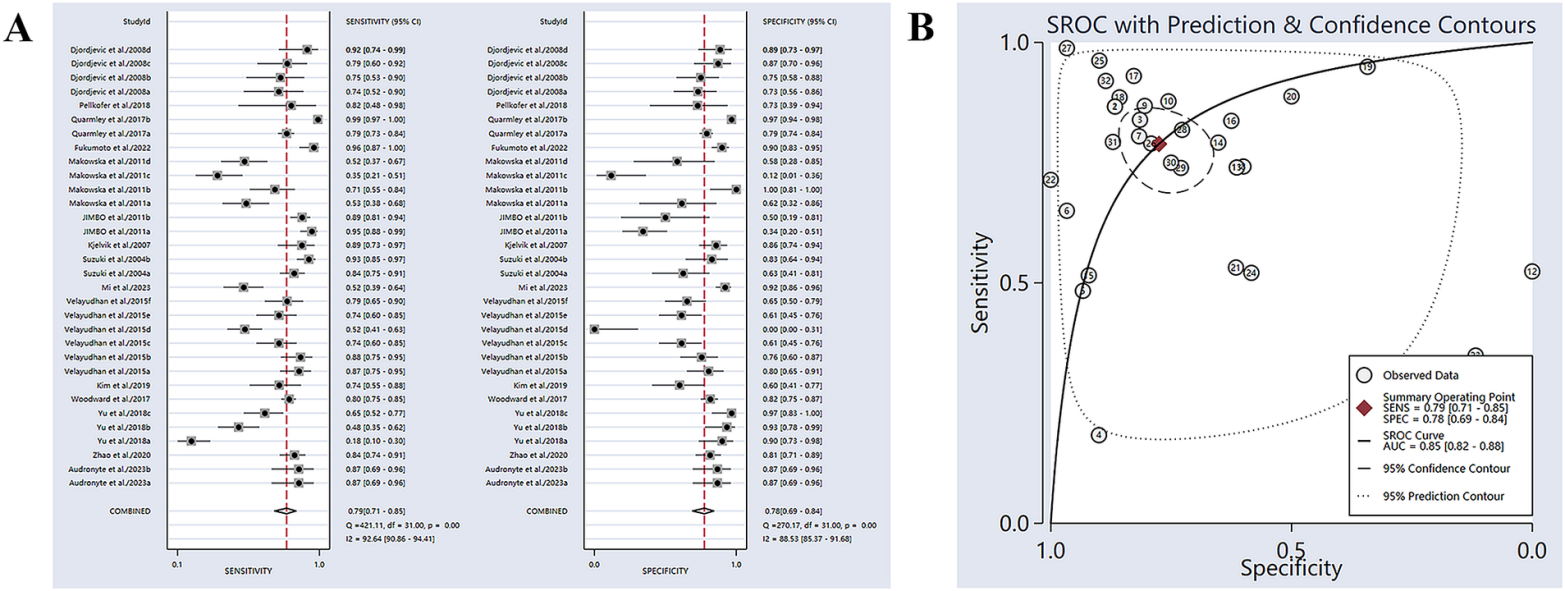
**(A)** SE and SP for diagnosing AD using olfactory function testing; **(B)** AUC for diagnosing AD using olfactory function testing.

Sixteen studies have reported the ability of olfactory function testing to discriminate healthy people and people with AD (Suzuki et al., 2004; Kjelvik et al., 2007; Djordjevic et al., 2008; Jimbo et al., 2011; Makowska et al., 2011; Velayudhan et al., 2015; Quarmley et al., 2017; Woodward et al., 2017; Yu et al., 2018; Kim et al., 2019; Pellkofer et al., 2019; Zhao et al., 2020; Dong et al., 2022; Fukumoto et al., 2022; Audronyte et al., 2023b; Mi et al., 2023). The SE and SP for diagnosing AD using olfactory function testing were 0.79 (95% CI [0.71–0.85], *I*^2^ = 92.64%) and 0.78 (95% CI [0.69–0.84], *I*^2^ = 88.53%), respectively (Figure 2A; Table 3). In addition, the pooled PLR and NLR were 3.51 (95% CI [2.44–5.06], *I*^2^ = 92.44%) and 0.27 (95% CI [0.19–0.39], *I*^2^ = 91.04%), respectively (Supplementary Figure S2A). The combined DOR was 12.90 (6.67–24.94, *I*^2^ = 100%) (Supplementary Figure S2B). The AUC was 0.85 (95% CI [0.82–0.88]) (Figure 2B; Table 3). Spearman’s correlation coefficient was −0.103 with a *p*-value of 0.573, indicating that there was no heterogeneity due to threshold effects.

**Table 3:** Diagnostic efficacy of olfactory function detection for diagnosing Healthy vs. AD, Healthy vs. MCI, MCI vs. AD, as well as olfactory function tests combined with other assessments for AD or MCI.

Outcome	SE (95%CI)	SP (95%CI)	AUC (95%CI)
Healthy vs. AD	0.79 (95% CI: 0.71–0.85)	0.78 (95% CI: 0.69–0.84)	0.85 (95% CI: 0.82–0.88)
Healthy vs. MCI	0.67 (95% CI: 0.54–0.78)	0.79 (95% CI: 0.71–0.86)	0.81 (95% CI: 0.77–0.84)
MCI vs. AD	0.58 (95% CI: 0.46–0.68)	0.88 (95% CI: 0.78–0.93)	0.78 (95% CI: 0.74–0.82)
Healthy vs. AD or MCI	0.83 (95% CI: 0.36–0.98)	0.94 (95% CI: 0.82–0.98)	0.96 (95% CI: 0.94–0.98)

#### 3.4.2 Subgroup analysis and regression analysis

Meta-regression and subgroup analyses were performed to analyze the sources of heterogeneity (Table 3). The year of publication was found to have an impact on heterogeneity (*p* = 0.02). Further analysis revealed that the combined SE of published studies after 2017 was significantly lower than those published before 2017 (>2017: 73%, CI: 55–86%; ≤2017: 81%, CI: 73–87%). There was no notable difference observed in the combined SE across different sample sizes (>100 and ≤100), different patient ages (>75 and ≤75), and different regions (Asia and non-Asia). There was no notable difference observed in the combined SP across different publication years (>2017 and ≤2017), different sample sizes (>100 and ≤100), different patient ages (>75 and ≤75), and regions of included participants (Asia and non-Asia).

#### 3.4.3 Clinical effect

According to Fagan’s nomogram, with a prior probability set at 50% (i.e., the probability of AD based on symptoms and signs was 50%), a positive diagnosis of olfactory function testing suggested a 78% probability of developing AD. If the diagnosis of olfactory function testing was negative, the probability of developing AD was 21% (Supplementary Figure S3A).

#### 3.4.4 Publication bias

Deek’s funnel plot asymmetry test was utilized to assess publication bias among the included studies (Supplementary Figure S4). The results showed significant publication bias (*p* < 0.01).

#### 3.4.5 Efficiency analysis of olfactory function testing for diagnosing healthy people and MCI

**Figure 3:**
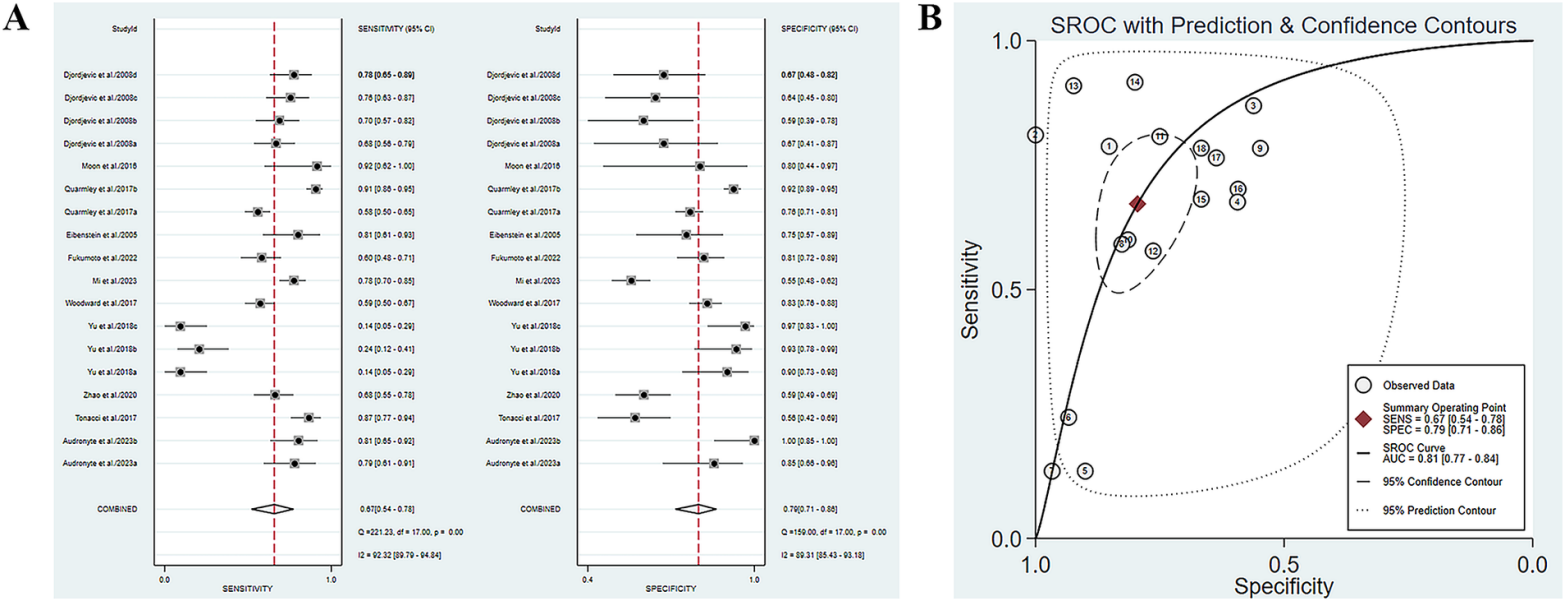
**(A)** SE and SP of MCI diagnosed by olfactory function testing; **(B)** AUC of MCI diagnosed by olfactory function testing.

Eleven studies have reported the ability of olfactory function testing to discriminate healthy people and MCI patients (Eibenstein et al., 2005; Djordjevic et al., 2008; Moon et al., 2016; Quarmley et al., 2017; Tonacci et al., 2017; Woodward et al., 2017; Yu et al., 2018; Zhao et al., 2020; Fukumoto et al., 2022; Audronyte et al., 2023b; Mi et al., 2023). The SE and SP of MCI diagnosed by olfactory function testing were 0.67 (95% CI [0.54–0.78], *I*^2^ = 92.32%) and 0.79 (95% CI [0.71–0.86], *I*^2^ = 89.31%), respectively (Figure 3A; Table 3). In addition, the pooled PLR and NLR were 3.28 (95% CI [2.35–4.57], *I*^2^ = 79.39%) and 0.41 (95% CI [0.29–0.58], *I*^2^ = 92.24%), respectively (Supplementary Figure S5A). The combined DOR was 7.95 (4.59–13.78, *I*^2^ = 100%) (Supplementary Figure S5B), and the AUC was 0.81 (95% CI [0.77–0.84]) (Figure 3B; Table 3). Spearman’s correlation coefficient was 0.236 with a *p*-value of 0.347, indicating that there was no heterogeneity due to threshold effects.

#### 3.4.6 Subgroup analysis and regression analysis

Meta-regression and subgroup analyses were conducted to analyze the sources of heterogeneity (Table 4). The sample size was found to have an impact on heterogeneity (*p* = 0.02). The combined SP of studies with sample sizes greater than 100 was significantly lower than that of studies with sample sizes less than or equal to 100 (75%, CI: 62–84% vs. 82%, CI: 72–90%). The average age of patients had an impact on heterogeneity (*p* = 0.01). The combined SP of studies with patient mean age greater than 72 years old was significantly lower than that of studies with patient mean age less than or equal to 72 years old (75%, CI: 65–83% vs. 85%, CI: 71–93%). The population included in the study had an impact on heterogeneity (*p* = 0.01). The combined SE of studies involving Asian populations was significantly lower than that of non-Asian populations (47%, CI: 25–70% vs. 76%, CI: 69–83%). There was no notable difference in the combined SE between different publication years (>2019 and ≤2019), different sample sizes (>100 and ≤100), and different patient ages (>75 and ≤75). There was no notable difference in the combined SP between different publication years (>2019 and ≤2019) and regions of included participants (Asia and non-Asia).

**Table 4:** Efficiency analysis of olfactory function testing for diagnosing healthy people and AD: meta-regression and subgroup analyses were performed to analyze the sources of heterogeneity.

Outcome	Subgroups		Sensitivity	Sensitivity-regression *p*	Specificity	Specificity-regression *p*	Positive likelihood ratio	Negative likelihood ratio	Diagnostic odds ratio	AUC
1-Distinguish AD from normal samples	Year of publication	>2017: 1	0.73 [0.55, 0.86]	0.02	0.87 [0.81, 0.92]	0.86	5.8 [3.8, 8.9]	0.31 [0.17, 0.54]	19 [8, 43]	0.89 [0.86, 0.92]
		≤2017: 0	0.81 [0.73, 0.87]		0.71 [0.59, 0.81]		2.8 [1.8, 4.3]	0.26 [0.17, 0.42]	11 [4, 25]	0.84 [0.80, 0.87]
	Sample size	>100: 1	0.89 [0.80, 0.94]	0.75	0.81 [0.68, 0.89]	0.29	4.6 [2.6, 8.3]	0.14 [0.07, 0.26]	34 [12, 100]	0.92 [0.89, 0.94]
		≤100: 0	0.72 [0.63, 0.79]		0.76 [0.65, 0.84]		3.0 [1.9, 4.7]	0.37 [0.26, 0.53]	8 [4, 17]	0.80 [0.76, 0.83]
	Mean age of patients	>75: 1	0.88 [0.83, 0.92]	0.95	0.80 [0.71, 0.86]	0.23	4.3 [2.9, 6.5]	0.15 [0.10, 0.23]	29 [14, 63]	0.91 [0.89, 0.93]
		≤75: 0	0.66 [0.55, 0.76]		0.76 [0.58, 0.87]		2.7 [1.4, 5.2]	0.45 [0.31, 0.67]	6 [2, 16]	0.75 [0.71, 0.79]
	Regions	Asia: 1	0.78 [0.62, 0.89]	0.06	0.81 [0.68, 0.90]	0.31	4.2 [2.5, 7.0]	0.27 [0.15, 0.47]	16 [8, 33]	0.87 [0.84, 0.90]
		Non- Asia: 0	0.79 [0.71, 0.85]		0.75 [0.65, 0.83]		3.2 [2.1, 4.9]	0.28 [0.18, 0.44]	11 [5, 26]	0.84 [0.80, 0.87]

#### 3.4.7 Clinical effect

According to Fagan’s nomogram, with a prior probability set at 50% (i.e., the probability of MCI based on symptoms and signs was 50%), a positive diagnosis of olfactory function testing suggested a 77% probability of developing MCI. If the diagnosis of olfactory function testing was negative, the probability of developing MCI was 29% (Supplementary Figure S3B).

#### 3.4.8 Publication bias

Deek’s funnel plot asymmetry test was utilized to assess publication bias among the included studies (Supplementary Figure S6). The results indicated that there was no significant publication bias (*p* = 0.59).

#### 3.4.9 Efficiency analysis of olfactory function testing for diagnosing MCI and AD

**Figure 4:**
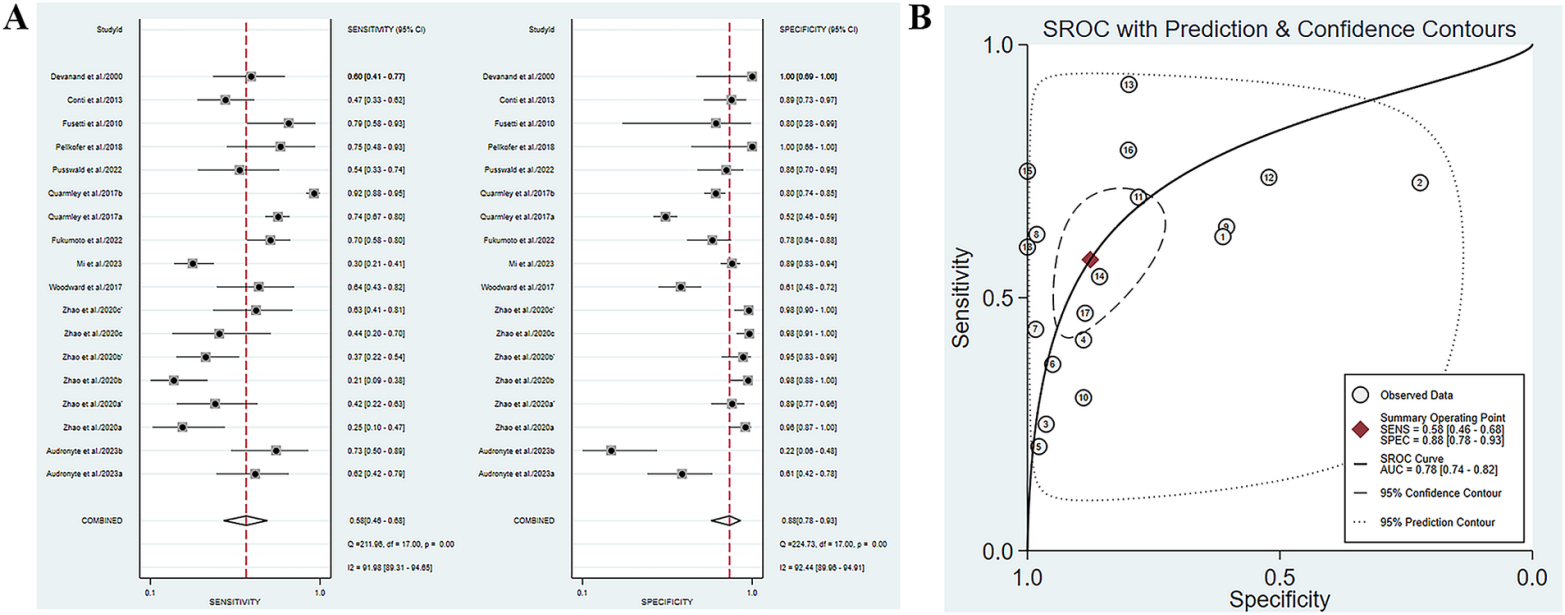
**(A)** SE and SP of olfactory function testing for the diagnosis of AD compared to MCI; **(B)** AUC of olfactory function testing for the diagnosis of AD compared to MCI.

Five studies have reported the ability of olfactory function testing to discriminate MCI and AD patients (Conti et al., 2013; Quarmley et al., 2017; Woodward et al., 2017; Pellkofer et al., 2019; Audronyte et al., 2023b). The SE and SP of olfactory function testing for the diagnosis of AD compared to MCI were 0.58 (95% CI [0.46–0.68], *I*^2^ = 91.98%) and 0.88 (95% CI [0.78–0.93], *I*^2^ = 92.44%), respectively (Figure 4A; Table 3). In addition, the pooled PLR and NLR were 4.62 (95% CI [2.66–8.02], *I*^2^ = 85.06%) and 0.49 (95% CI [0.39–0.61], *I*^2^ = 91.26%), respectively (Supplementary Figure S7A). The combined DOR was 9.52 (5.08–17.84, *I*^2^ = 100%) (Supplementary Figure S7B), and the AUC was 0.78 (95% CI [0.74–0.82]) (Figure 4B; Table 3). Spearman’s correlation coefficient was 0.589 with a *p*-value of 0.021, indicating the presence of heterogeneity caused by threshold effects (Table 5).

**Table 5:** Efficiency analysis of olfactory function testing for diagnosing healthy people and MCI: meta-regression and subgroup analyses were conducted to analyze the sources of heterogeneity.

Outcome	Subgroups	Sensitivity	Sensitivity-regression *p*	Specificity	Specificity-regression *p*	Positive likelihood ratio	Negative likelihood ratio	Diagnostic odds ratio	AUC	AUC
2-Distinguish MCI from normal samples	Year of publication	>2019: 1	0.73 [0.65, 0.80]	0.97	0.80 [0.57, 0.92]	0.12	3.7 [1.5, 7.2]	0.34 [0.23, 0.50]	11 [3, 18]	0.79 [0.75, 0.82]
		≤2019: 0	0.65 [0.46, 0.79]		0.80 [0.71, 0.87]		3.3 [2.3, 4.7]	0.44 [0.29, 0.68]	7 [4, 14]	0.81 [0.77, 0.84]
	Sample size	>100: 1	0.74 [0.62, 0.83]	0.89	0.75 [0.62, 0.84]	0.02	2.9 [1.8, 4.7]	0.35 [0.23, 0.55]	8 [4, 19]	0.81 [0.77, 0.84]
		≤100: 0	0.62 [0.42, 0.78]		0.82 [0.72, 0.90]		3.5 [2.3, 5.4]	0.46 [0.30, 0.72]	8 [4, 15]	0.82 [0.78, 0.85]
	Mean age of patients	>72: 1	0.74 [0.64, 0.81]	0.72	0.75 [0.65, 0.83]					
	0.01	2.9 [2.0, 4.3]	0.35 [0.25, 0.51]	8 [4, 16]	0.81 [0.77, 0.84]					
		≤72: 0	0.59 [0.35, 0.79]		0.85 [0.71, 0.93]		3.9 [2.1, 7.4]	0.48 [0.28, 0.82]	8 [3, 21]	0.82 [0.79, 0.86]
	Regions	Asia: 1	0.47 [0.25, 0.70]	0.01	0.82 [0.65, 0.92]	0.27	2.6 [1.8, 3.9]	0.65 [0.48, 0.89]	4 [3, 6]	

#### 3.4.10 Clinical effect

According to Fagan’s nomogram, with a prior probability set at 50% (i.e., the probability of AD based on symptoms and signs was 50%), a positive diagnosis of olfactory function testing suggested an 82% probability of developing AD, compared to MCI. If the diagnosis of olfactory function testing was negative, the probability of developing AD was 33% (Supplementary Figure S3C).

#### 3.4.11 Publication bias

Deek’s funnel plot asymmetry test was utilized to assess publication bias among the included studies (Supplementary Figure S8). The results indicated that there was no significant publication bias (*p* = 0.87).

#### 3.4.12 Efficiency analysis of olfactory function testing combined with other tests for diagnosing AD or MCI

**Figure 5:**
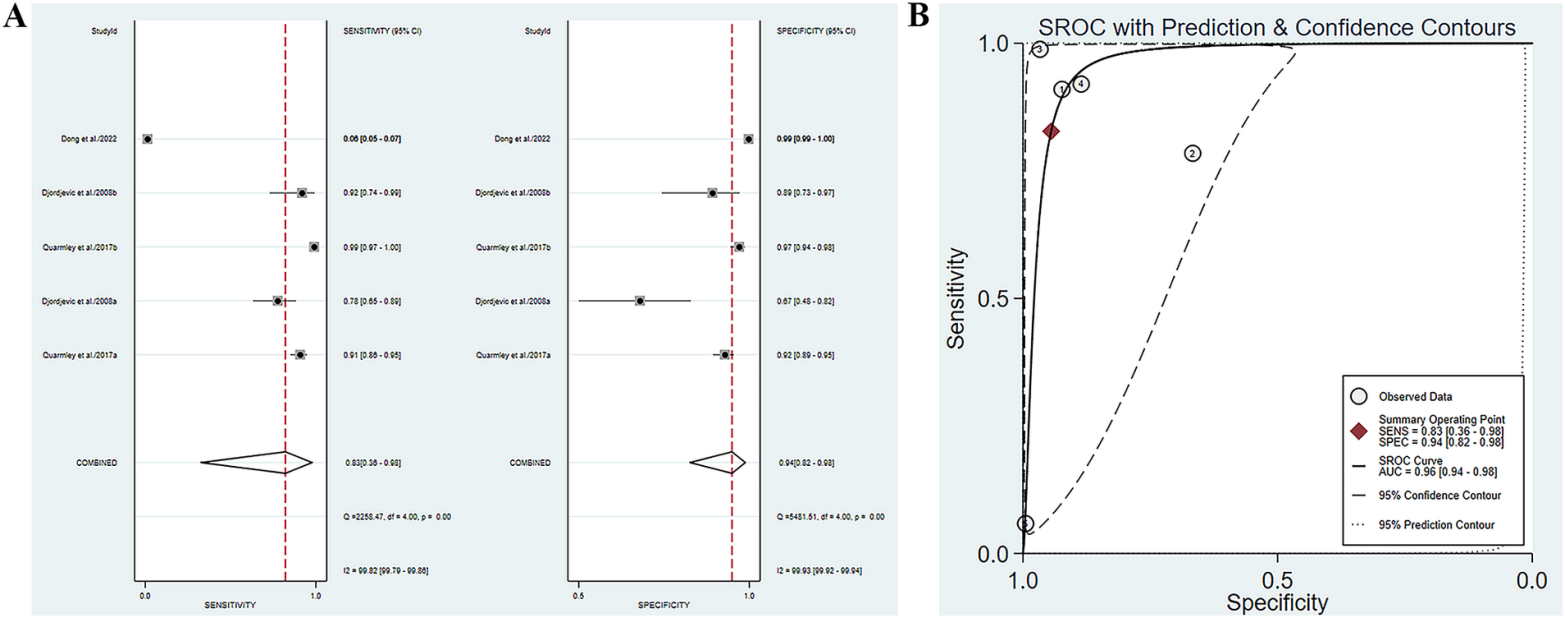
**(A)** SE and SP of olfactory function testing combined with other indicators for diagnosing AD or MCI; **(B)** AUC of olfactory function testing combined with other indicators for diagnosing AD or MCI.

There are three studies reporting the combination of olfactory function testing with other methods (SS-OIT combined with MoCa scale, SIT combined with computer optimal formula, SSIT combined with AD8 self-assessment questionnaire) for the diagnosis of MCI or AD (Djordjevic et al., 2008; Quarmley et al., 2017; Dong et al., 2022). The SE and SP of olfactory function testing combined with other indicators for diagnosing AD or MCI were 0.83 (95% CI [0.36–0.98], *I*^2^ = 99.82%) and 0.94 (95% CI [0.82–0.98], *I*^2^ = 99.93%), respectively (Figure 5A; Table 3). In addition, the pooled PLR and NLR were 14.98 (95% CI [4.92–45.66], *I*^2^ = 99.59%) and 0.18 (95% CI [0.03–1.05], *I*^2^ = 99.98%), respectively (Supplementary Figure S9A). The combined DOR was 82.09 (12.92–521.48, *I*^2^ = 100%) (Supplementary Figure S9B), and the AUC was 0.96 (95% CI [0.94–0.98]) (Figure 5B; Table 3). Spearman’s correlation coefficient was 0.1 with a *p*-value of 0.873, indicating that there was no heterogeneity due to threshold effects.

#### 3.4.13 Clinical effect

According to Fagan’s nomogram, with a prior probability set at 50% (i.e., the probability of AD or MCI based on symptoms and signs was 50%), a positive diagnosis of olfactory function testing combined with other tests suggested a 94% probability of developing AD or MCI. If the diagnosis of olfactory function testing combined with other tests was negative, the probability of developing AD or MCI was 15% (Supplementary Figure S3D).

#### 3.4.14 Publication bias

Deek’s funnel plot asymmetry test was utilized to assess publication bias among the included studies (Supplementary Figure S10). The results indicated that there was no significant publication bias (*p* = 0.41).

#### 3.4.15 Relationship between olfactory function and progression to MCI and AD in normal individuals

Four studies reported the impact of olfactory function on the progression of MCI and AD in normal individuals (Jimbo et al., 2011; Liang et al., 2016; Churnin et al., 2019; Mi et al., 2023). Meta-analysis was conducted using a random-effects model (*I*^2^ = 87.4%, *p* < 0.001). The results showed that a decline in olfactory function was a risk factor for cognitive impairment in normal individuals (OR = 1.83, 95% CI [1.41–2.38], *p* < 0.01) (Supplementary Figure S11A).

#### 3.4.16 Relationship between olfactory function and progression of MCI to AD patients

Three studies reported the impact of olfactory function on patients with MCI progressing to AD (Zhao et al., 2020; Pusswald et al., 2022; Mi et al., 2023). Meta-analysis was conducted using a random-effects model (*I*^2^ = 67.3%, *p* = 0.016). The results indicated a significant association between the decline of olfactory function and the progression of MCI to AD patients. The risk of progression from MCI to AD increased as olfactory function declined more severely (OR = 1.52, 95% CI [1.16–1.99], *p* = 0.003) (Supplementary Figure S11B).

## 4 Discussion

Diagnostic or correlation data between olfactory function testing and AD and MCI were analyzed through a systematic review of existing studies. The results indicated a correlation between decreased olfactory function and the occurrence of AD and MCI. By pooling the AUC values, olfactory function testing demonstrated effective diagnostic performance for AD and MCI. Moreover, the combined diagnosis of olfactory function testing and other examination methods had more excellent diagnostic performance. In terms of correlation, decreased olfactory function was identified as a risk factor for the development of cognitive impairment in normal individuals. In addition, there was a significant correlation observed between decreased olfactory function and the progression from MCI to AD in patients.

### 4.1 Heterogeneity discussion

In distinguishing MCI from AD, Spearman’s correlation coefficient was 0.589 (*p* = 0.021), suggesting that the heterogeneity mainly arises from the cutoff values. Variations in olfactory detection methods and cutoff values across studies could be key factors leading to the differences in results. Olfactory detection methods (such as the UPSIT and SS-OIT) and their cutoff values (i.e., the standards employed to determine normal or abnormal olfactory function) vary due to differences in study design and sample populations. For example, in the study by Woodward et al., the cutoff value for the top 10 items on the UPSIT is established at 7 (SE: 74%, SP: 71%) [28243501]. In the study by Quarmley et al., the cutoff value for the SS-OIT is set at 9 (SE: 58%, SP: 69%) [27886011]. According to Conti et al., the cutoff value for the CA-SIT Smell Identification Test is identified as 24 (SE: 47%, SP: 89%) [23669447]. Furthermore, in the study by Devanand et al., the cutoff value for the UPSIT is defined as 34 (SE: 90%, SP: 50%) [10964854]. The differences in detection methods and cutoff values may impact the SE and SP of diagnoses, thereby leading to inconsistencies in research findings. Particularly in the early diagnosis of AD or MCI, variations in the cutoff values for olfactory tests may lead to an erroneous classification of some patients’ olfactory function as normal, thereby impacting the early detection of these conditions. Therefore, when conducting cross-study comparisons, it is essential to take these methodological differences into account and explicitly mention them in the research report to facilitate a more accurate interpretation of the results.

Due to significant heterogeneity, subgroup analysis was conducted to identify the source of heterogeneity in the study. The year of publication, sample size included, average age of patients, and region of study participants were found to be factors affecting heterogeneity.

In terms of discriminating between healthy people and AD patients, articles published before 2017 demonstrated significantly higher SE in diagnosing AD compared to those published after 2017. Early studies may have utilized different diagnostic criteria that potentially favored higher SE. New diagnostic criteria are more stringent and refined, focusing more on a comprehensive assessment rather than a single indicator, leading to a reduction in SE. Moreover, early studies may have selected more typical cases of AD, while later studies included more early and mild cases for better early diagnosis, resulting in decreased SE.

In terms of discriminating between healthy people and MCI patients, studies with sample sizes equal to or less than 100 demonstrated significantly higher SE compared to studies with sample sizes greater than 100. Studies with an average patient age over 72 years old indicated significantly lower SE compared to studies with an average patient age of 72 years old or younger. Small sample size studies can better control variables, resulting in higher SE and greater susceptibility to sample selection bias. Patients over 72 years of age may have more extensive cognitive decline with increasing age. Moreover, these patients may have more comorbidities with other diseases, increasing the complexity of the diagnosis. The complexity of conditions makes it more difficult to differentiate between MCI and other cognitive disorders, leading to reduced SE.

In terms of discriminating MCI and AD patients, studies with an average patient age older than 68 years old exhibited significantly higher SE compared to studies with an average patient age of 68 years old or younger. However, SP showed the opposite trend. Older patients may have entered the distinctly olfactory stage of AD, with higher SE but lower SP. In younger patients, the olfactory symptoms of MCI may be atypical, resulting in lower SE but higher SP.

The SE in the Asian population showed a notable decrease compared to the non-Asian population in distinguishing healthy people and MCI patients and distinguishing MCI and AD patients. SE is influenced by racial factors, possibly due to differences in prevalent diagnostic methods in the region. It may be affected if some countries modify the original diagnostic method to be more appropriate for their population. It also may be related to that there are certain differences in olfactory SE among different races. Common olfactory detection methods include UPSIT, SS-OIT, B-SIT, San Diego odor identification test, and odor stick identification test for Japanese. These methods typically assess odor recognition, discrimination, and threshold levels, contributing to a comprehensive evaluation of olfactory function and aiding in the determination of potential causes for functional decline [36768440]. If there is polymorphism in the olfactory receptor gene in the genetic background, it can lead to differences in olfactory SE (Ignatieva et al., 2014). With different cultures and dietary habits, people exposed to specific odors for a long time may be more sensitive. In addition, people from different cultural backgrounds may have varying perceptions and descriptions of certain odors (Pinto et al., 2014). These differences can affect the results of the olfactory testing. Environmental exposures such as air quality, climate, and pollution levels in both developing and developed countries can also have an impact on olfactory function.

### 4.2 Limitations and strengths

Although this study provided valuable insights, there were some limitations. Firstly, the heterogeneity of the included studies may affect the interpretation of results. Factors such as methods of olfactory testing used in different studies, population characteristics of participants, and severity of condition may have contributed to the differences in results. Secondly, causality cannot be determined due to the study design, which was mainly based on cross-sectional studies. More longitudinal studies are needed to further validate the predictive role of olfactory impairment in the progression of AD and MCI. In addition, olfactory function may be influenced by multiple factors such as age, gender, and smoking history, which need to be controlled in future studies. Finally, all included studies were published in English, which may result in selection bias.

Based on the findings of this study, future studies should further explore the application effects of olfactory function testing in various populations of different ages, genders, and races. In addition, the longitudinal study design will help determine the causal relationship between olfactory impairment and the progression of AD and MCI. Based on comprehensive studies of other cognitive-related scales or questionnaires, the results showed that the AUC of the combined diagnosis of AD/MCI was 0.96, indicating good diagnostic performance. According to meta-analyses of traditional laboratory methods for diagnosing AD/MCI, the AUC values were as follows: 0.878 for RNA sequencing diagnosis, 0.90 for CSF testing diagnosis AUC of, and 0.93 for clinical symptom scale diagnosis (Shigemizu et al., 2020; Burgio et al., 2024). These data also indicated that the diagnostic performance of olfactory testing combined with cognitive-related scales or questionnaires has been improved compared to individual testing. Moreover, compared to laboratory tests, combined tests are non-invasive, easy to perform, and cost-effective. However, due to the limited amount of available literature related to the diagnosis of olfactory function testing combined with cognitive scales, more clinical studies are needed to confirm this viewpoint.

### 4.3 Future prospects

First, promoting international cooperation and establishing globally unified olfactory testing standards can ensure the comparability of study results across different regions. Furthermore, diagnostic criteria can also be adjusted based on different populations and cultural backgrounds. For example, testing for different populations should consider their common olfactory experiences and habits. Secondly, it is necessary to increase multi-center large sample studies to obtain more representative data and reduce bias caused by small sample studies. Studies should cover populations of different age groups, genders, races, and educational levels to improve the generalizability of the results. In addition, it is necessary to strictly control variables and reduce the interference of external factors on the results, making the results more scientific and reliable. A prospective design should be adopted to further enhance the credibility of the study results. In terms of early screening, olfactory testing tools should be developed for different age groups. For young patients, it can improve the early detection rate. In high-risk populations, early screening is promoted with the goal of timely detection and intervention for cognitive impairments. By conducting reasonable publicity, public awareness of olfactory testing in the diagnosis of AD and MCI can be enhanced. Promoting early screening programs in the community and primary care settings and encouraging early and regular screening in at-risk populations may have a positive impact on early detection of AD and MCI.

The comprehensive evaluation of olfactory testing and other examination methods combined in the results section of the article can help improve diagnostic accuracy. The combined results section showed that olfactory testing in combination with other screening methods improves diagnostic accuracy. This suggests that combined assessment has a positive effect on improving diagnosis. The diagnosis of AD and MCI can be comprehensively evaluated by combining various methods such as neuroimaging, biomarkers, and olfactory testing, and establishing a dynamic detection system. Long-term follow-up of patients is also needed to adjust the diagnosis and treatment plan in time. Moreover, personalized diagnosis and treatment plans can be developed based on the patient’s living environment and habits.

With such measures, we believe that the SE and SP of olfactory testing in the diagnosis of AD and MCI can be improved to a certain degree. As a result, earlier and more effective interventions and treatments for patients can be realized, thus improving the overall standard of care.

## 5 Conclusion

This meta-analysis suggests that olfactory impairment is common among patients with AD and MCI, showing a strong association with the early stages of these conditions. Olfactory function testing, as a simple and low-cost tool, has the potential for early screening and diagnosis of AD and MCI. This discovery provides a new perspective for the application of olfactory impairment in neurodegenerative diseases. Moreover, it provides an important reference for future research and clinical practice.

## Data Availability

The original contributions presented in the study are included in the article/Supplementary material, further inquiries can be directed to the corresponding authors.
