# Supplementary material for: Diagnostic value of olfactory function testing for Alzheimer’s disease and mild cognitive impairment: a systematic review and meta-analysis
Source: Front Aging Neurosci. 2025 May 15;17:1551939. doi: 10.3389/fnagi.2025.1551939 (PMC12119486; doi:10.3389/fnagi.2025.1551939)
Supplement: Supplementary file 1 [file Table_1.docx]

Supplementary Material

**Diagnostic Value of Olfactory Function Testing for Alzheimer’s Disease and Mild Cognitive Impairment: A systematic review**

**Short title:** Olfactory Testing for AD and MCI

**Yuxuan Liu^1^, Yunpeng Cao^2*^, Hongquan Wei^1*^**

^1^Department of Otolaryngology, the First Affiliated Hospital of China Medical University, Shenyang, China

^2^Department of Neurology, the First Affiliated Hospital of China Medical University, Shenyang, China

*Hongquan Wei and Yunpeng Cao should be considered as co-corresponding authors, and Hongquan Wei is the first corresponding author.

* Correspondence:
Hongquan Wei
Email: [hongquanwei@163.com](mailto:hongquanwei@163.com)
Yunpeng Cao
Email: [cypcmu@163.com](mailto:cypcmu@163.com)

# File S1 Literature search strategy

**1.Pubmed**

| Search number | Query |
| --- | --- |
| #1 | "Alzheimer Disease"[Mesh] |
| #2 | "Alzheimer Disease"[Title/Abstract] OR "alzheimer*"[Title/Abstract] OR "dementia*"[Title/Abstract] |
| #3 | #1 OR #2 |
| #4 | "Cognitive Dysfunction"[Mesh] |
| #5 | "mild Cognitive Dysfunction*"[Title/Abstract] OR "mild cognitive impair*"[Title/Abstract] OR "mild cognitive disorder*"[Title/Abstract] OR "mild cognitive disability"[Title/Abstract] OR "mild cognitive deficit"[Title/Abstract] OR "mild cognitive defect*"[Title/Abstract] OR "mild mental deterioration*"[Title/Abstract] |
| #6 | #4 OR #5 |
| #7 | #3 OR #6 |
| #8 | "Olfaction Disorders"[Mesh] |
| #9 | "Olfaction Disorders"[Title/Abstract] OR "olfaction disorder*"[Title/Abstract] OR "smelling disorder*"[Title/Abstract] OR "olfact* impairment*"[Title/Abstract] OR "olfact* dysfunction"[Title/Abstract] OR "olfact* anomaly"[Title/Abstract] OR "olfact* disturbance"[Title/Abstract] OR "olfact* diseas*"[Title/Abstract] OR "paraosmia*"[Title/Abstract] OR "phantosmia*"[Title/Abstract] OR "parosmia*"[Title/Abstract] OR "impaired olfaction*"[Title/Abstract] OR "cacosmia*"[Title/Abstract] OR "dysosmia*"[Title/Abstract] |
| #10 | #8 OR #9 |
| #11 | "Nose"[Mesh] |
| #12 | (nasal[Title/Abstract]) OR (Nose[Title/Abstract]) |
| #13 | #11 OR #12 |
| #14 | ("Proteins"[Mesh]) OR "Biomarkers"[Mesh] |
| #15 | "Protein*"[Title/Abstract] OR "biomarker*"[Title/Abstract] OR "amyloid beta"[Title/Abstract] OR "alpha-Synuclein"[Title/Abstract] OR "beta amyloid aggregates"[Title/Abstract] OR "alpha synuclein aggregates"[Title/Abstract] OR "Aβ"[Title/Abstract] OR "Tau"[Title/Abstract] OR "marker*"[Title/Abstract] OR "enzyme*"[Title/Abstract] OR "metabolite*"[Title/Abstract] OR "hormon*" OR[Title/Abstract] |
| #16 | #14 OR #15 |
| #17 | "Diagnosis"[Mesh] |
| #18 | "Prognosis"[Mesh] |
| #19 | "diagnos*"[Title/Abstract] OR "prognos*"[Title/Abstract] OR "forecast*"[Title/Abstract] OR "predict*"[Title/Abstract] OR "AUC"[Title/Abstract] OR "ROC"[Title/Abstract] OR "accuracy"[Title/Abstract] OR "receiver operator*"[Title/Abstract] OR "sensitivity"[Title/Abstract] OR "specificity"[Title/Abstract] |
| #20 | #17 OR #18 OR #19 |
| #21 | #13 AND #16 |
| #22 | #21 OR #10 |
| #23 | #7 AND #20 AND #22 |

**2.Cochrane**

| Search number | Query |
| --- | --- |
| #1 | MeSH descriptor: [Alzheimer Disease] explode all trees |
| #2 | (‘Alzheimer Disease’ OR ‘alzheimer*’ OR ‘dementia*’) |
| #3 | #1 or #2 |
| #4 | MeSH descriptor: [Cognitive Dysfunction] explode all trees |
| #5 | (mild Cognitive Dysfunction*):ti,ab,kw OR (mild cognitive impair*):ti,ab,kw OR (mild cognitive disorder*):ti,ab,kw OR (mild cognitive disability):ti,ab,kw OR (mild cognitive deficit):ti,ab,kw |
| #6 | (mild cognitive defect*):ti,ab,kw OR (mild mental deterioration*):ti,ab,kw |
| #7 | #6 or #5 |
| #8 | #7 or #4 |
| #9 | #8 or #3 |
| #10 | MeSH descriptor: [Olfaction Disorders] explode all trees |
| #11 | (‘Olfaction Disorders’ OR ‘olfaction disorder*’ OR ‘smelling disorder*’ OR ‘olfact* impairment*’ OR ‘olfact* dysfunction’ OR ‘olfact* anomaly’ OR ‘olfact* disturbance’ OR ‘olfact* diseas*’ OR ‘paraosmia*’ OR ‘phantosmia*’ OR ‘parosmia*’ OR ‘impaired olfaction*’ OR ‘cacosmia*’ OR ‘dysosmia*’):ti,ab,kw |
| #12 | #10 OR #11 |
| #13 | MeSH descriptor: [Nose] explode all trees |
| #14 | (‘Nose’ OR ‘nasal’):ti,ab,kw |
| #15 | #13 or #14 |
| #16 | MeSH descriptor: [Proteins] explode all trees |
| #17 | MeSH descriptor: [Biomarkers] explode all trees |
| #18 | (‘Protein*’ OR ‘biomarker*’ OR ‘amyloid beta’ OR ‘alpha-Synuclein’ OR ‘beta amyloid aggregates’ OR ‘alpha synuclein aggregates’ OR ‘Aβ’ OR ‘Tau’ OR ‘marker*’ OR ‘enzyme*’ OR ‘metabolite*’ OR ‘hormon*’):ti,ab,kw |
| #19 | #16 or #17 or #18 |
| #20 | MeSH descriptor: [Diagnosis] explode all trees |
| #21 | MeSH descriptor: [Prognosis] explode all trees |
| #22 | (‘diagnos*’ OR ‘prognos*’ OR ‘forecast*’ OR ‘predict*’ OR ‘AUC’ OR ‘ROC’ OR ‘accuracy’ OR ‘receiver operator*’ OR ‘sensitivity’ OR ‘specificity’):ti,ab,kw |
| #23 | #20 or #21 or #22 |
| #24 | #15 and #19 |
| #25 | #24 or #12 |
| #26 | #9 and #23 and #25 |

**3.Embase**

| Search number | Query |
| --- | --- |
| #1 | 'alzheimer disease'/exp |
| #2 | 'alzheimer disease':ti,ab,kw OR 'alzheimer*':ti,ab,kw OR 'dementia*':ti,ab,kw |
| #3 | #1 OR #2 |
| #4 | 'cognitive defect'/exp |
| #5 | 'mild cognitive dysfunction*':ti,ab,kw OR 'mild cognitive impair*':ti,ab,kw OR 'mild cognitive disorder*':ti,ab,kw OR 'mild cognitive disability':ti,ab,kw OR 'mild cognitive deficit':ti,ab,kw OR 'mild cognitive defect*':ti,ab,kw OR 'mild mental deterioration*':ti,ab,kw |
| #6 | #4 OR #5 |
| #7 | #3 OR #6 |
| #8 | 'smelling disorder'/exp |
| #9 | 'olfaction disorders':ti,ab,kw OR 'olfaction disorder*':ti,ab,kw OR 'smelling disorder*':ti,ab,kw OR 'olfact* impairment*':ti,ab,kw OR 'olfact* dysfunction':ti,ab,kw OR 'olfact* anomaly':ti,ab,kw OR 'olfact* disturbance':ti,ab,kw OR 'olfact* diseas*':ti,ab,kw OR 'paraosmia*':ti,ab,kw OR 'phantosmia*':ti,ab,kw OR 'parosmia*':ti,ab,kw OR 'impaired olfaction*':ti,ab,kw OR 'cacosmia*':ti,ab,kw OR 'dysosmia*':ti,ab,kw |
| #10 | #8 OR #9 |
| #11 | 'nose'/exp |
| #12 | 'nose':ti,ab,kw OR 'nasal':ti,ab,kw |
| #13 | #11 OR #12 |
| #14 | 'protein'/exp OR 'biomarker'/exp |
| #15 | 'protein*':ti,ab,kw OR 'biomarker*':ti,ab,kw OR 'amyloid beta':ti,ab,kw OR 'alpha-synuclein':ti,ab,kw OR 'beta amyloid aggregates':ti,ab,kw OR 'alpha synuclein aggregates':ti,ab,kw OR 'aβ':ti,ab,kw OR 'tau':ti,ab,kw OR 'marker*':ti,ab,kw OR 'enzyme*':ti,ab,kw OR 'metabolite*':ti,ab,kw OR 'hormon*':ti,ab,kw |
| #16 | #14 OR #15 |
| #17 | 'diagnosis'/exp |
| #18 | 'prognosis'/exp |
| #19 | 'diagnos*':ti,ab,kw OR 'prognos*':ti,ab,kw OR 'forecast*':ti,ab,kw OR 'predict*':ti,ab,kw OR 'auc':ti,ab,kw OR 'roc':ti,ab,kw OR 'accuracy':ti,ab,kw OR 'receiver operator*':ti,ab,kw OR 'sensitivity':ti,ab,kw OR 'specificity':ti,ab,kw |
| #20 | #17 OR #18 OR #19 |
| #21 | #13 AND #16 |
| #22 | #10 OR #21 |
| #23 | #7 AND #20 AND #22 |

**4.Web of science**

| Search number | Query |
| --- | --- |
| #1 | "Alzheimer Disease (Topic) OR alzheimer* (Topic) OR dementia* (Topic) OR mild Cognitive Dysfunction* (Topic) OR mild cognitive impair* (Topic) OR mild cognitive disorder* (Topic) OR mild cognitive disability (Topic) OR mild cognitive deficit (Topic) OR mild cognitive defect* (Topic) OR mild mental deterioration* (Topic) OR Cognitive Dysfunction (Topic) and Preprint Citation Index (Exclude – Database)" |
| #2 | "Olfaction Disorders (Topic) OR Olfaction Disorders (Topic) OR olfaction disorder* (Topic) OR smelling disorder* (Topic) OR olfact* impairment* (Topic) OR olfact* dysfunction (Topic) OR olfact* anomaly (Topic) OR olfact* disturbance (Topic) OR olfact* diseas* (Topic) OR paraosmia* (Topic) OR phantosmia* (Topic) OR parosmia* (Topic) OR impaired olfaction* (Topic) OR cacosmia* (Topic) OR dysosmia* (Topic) OR Olfaction Disorders (Topic) and Preprint Citation Index (Exclude – Database " |
| #3 | "Nose (Topic) OR nasal (Topic) and Preprint Citation Index (Exclude – Database)" |
| #4 | "diagnos* (Topic) OR prognos* (Topic) OR forecast* (Topic) OR predict* (Topic) OR AUC (Topic) OR ROC (Topic) OR accuracy (Topic) OR receiver operator* (Topic) OR receiver operator* (Topic) OR specificity (Topic) and Preprint Citation Index (Exclude – Database)" |
| #5 | "Protein* (Topic) OR biomarker* (Topic) OR amyloid beta (Topic) OR alpha-Synuclein (Topic) OR beta amyloid aggregates (Topic) OR alpha synuclein aggregates (Topic) OR Aβ (Topic) OR Tau (Topic) OR marker* (Topic) OR enzyme* (Topic) OR metabolite* (Topic) OR hormon* (Topic) and Preprint Citation Index (Exclude – Database)" |
| #6 | "#5 AND #3 and Preprint Citation Index (Exclude – Database) " |
| #7 | "#6 AND #4 AND #2 AND #1 and Preprint Citation Index (Exclude – Database)" |

**File S2** A detailed overview of the basic characteristics of the included studies

| No. | First Author | Publication Year | Country | Study Design  (case control/cross-sectional/cohort/prospective study) | Diagnosis or prognosis | N  (Male/Female) | Age  Mean±SD or Median(IQR) or Median[range] or range | Testing methods | Assessment of Cognitive Functions | Outcome | Main index |
| --- | --- | --- | --- | --- | --- | --- | --- | --- | --- | --- | --- |
| 1 | Audronyte et al. | 2023 | Lithuania | case control study | Diagnosis | Normal cognition: 30 (13/17)  MCI-AD: 30 (13/17)  MD (mild dementia)-AD: 30 (12/18) | Normal cognition:74 (68.75, 76)  MCI-AD: 72 (67.75, 77.25)  MD-AD: 78 (75, 79.25) | (1) Sniffin’ Sticks odor identification test  (2) odor discrimination test | (1) MiniMental State Examination (MMSE)  (2) CDR  (3) Alzheimer’s Disease Assessment Scale-Cognitive Subscale (ADAS-Cog)  (4) Phonemic (PAS) and categorical (animals) verbal fluency | AD  MCI-AD  MD-AD | sensitivity and specificity  AUC |
| 2 | Tonacci et al. | 2017 | Italy | cross sectional study | Diagnosis | MCI: 85 (41/44)  Controls: 41 (22/19) | MCI: 76±4.9  Controls: 73.5±4.3 | (1) Sniffin‘ticks Extended Test  (2) TDI Score | (1) ADAS-cog (Alzheimer's  Disease Assessment Scale-cognitive subscale)  (2) ADL (Activities of Daily Living)  (3) CDR (Clinical Dementia Rating)  (4) CDT (Clock Drawing Test)  (5) MMSE (Mini-Mental State  Examination)  (6) RAVLT (Rey Auditory Verbal Learning Test)  (7) ROCF (Rey–Osterrieth complex figure test)  (8) Word Recognition  (9) Digit Span  (10) Attentional Matrices  (11) Immediate proserecall  (12) Delayed proserecall  (13) Free drawing copy test  (14) Programmed drawing copy test  (15) Raven's progressice matrices  (16) Verbal fluency  (17) Semantic verbal fluency  (18) Span Corsiblock-tapping test | MCI | sensitivity and specificity  AUC |
| 3 | Zhao et al. | 2020 | China | cohort study  cross sectional study | Diagnosis  Prognosis | Baseline：  HC (Health Control): 80 (36/44)  MCI: 87 (40/47)  AD dementia: 88 (38/50)  2-year follow-up：baseline MCI (78)  Non-progression: 70 (NA)  Progression to AD: 8 (NA)  3-year follow-up：baseline MCI (78)  Non-progression: 62 (28/34)  Progression to AD: 16 (7/9) | Baseline：  HC (Health Control): 67.3±4.7  MCI: 66.2±4.3  AD dementia: 67.7±4.2  2-year follow-up: baseline MCI (NA)  3-year follow-up: baseline MCI  Non-Progression to MCI: 68.3±4.1  Progression to MCI: 68.5±3.8 | (1) SS-16 assessment | (1) MMSE  (2) MoCA  (3) SAS (Zung Self-rating the Anxiety Scale)  (4) SDS (Zung Self-rating Depression Scale, ADAS-cog Alzheimer’s Disease Assessment Scale-cognitive subscale)  (5) ADAS-cog  (6) AVLT-SR (Auditory Verbal Learning test)  (7) AVLT-LR (Auditory Verbal Learning test) | AD | AUC  sensitivity  specificity |
| 4 | Yu et al. | 2018 | China | cross sectional study | Diagnosis | Control Group: 30 (10/20)  MCI: 37 (10/27)  AD: 60 (24/36) | Control Group: 62.00 (60.00, 69.50)  MCI: 64.00 (58.50, 71.00)  AD: 64.00 (58.50, 71.00) | (1) Sniffin' Sticks test  (2) HRS  (3) Self-Report | (1) MMSE  (2) MoCA  (3) AVLT  (4) CFT (Complex Figure Test)-delayed memory  (5) AFT (Animal Fluency Test)  (6) TMT-A (Trail Making Test A)  (7) SCWT (Stroop Color-Word Test)  (8) TMT-B (Trail Making Test B) | MCI  AD | sensitivity  specificity |
| 5 | Woodward et al. | 2017 | America | cross sectional study  cohort study | Diagnosis  Prognosis | Normal control: 194 (59/135)  MCI: 110 (57/53)  AD: 262 (131/131)  2-year follow-up：  25 of 96 patients with MCI progressed to AD. | Normal control: 72.29±8.41  MCI: 74.05±9.03  AD: 75.62±8.29 | (1) UPSIT(University of Pennsylvania Smell Identification Test) | (1) MMSE  (2) Clinical Dementia Rating  (3) Digit Span  (4) Trail Making Test Part A  (5) Trail Making Test Part B  (6) WMS3 or R (Wechsler Memory Scale)  (7) Boston Naming Test and FAS Verbal Fluency  (8) American National Adult Reading Test  (9) WMS–Visual Reproduction I and II  (10) Geriatric Depression Scale  (11) Lawton-Brody Activities of Daily Living  (12) Physical Self-Maintenance Scale  (13) Instrumental Activities of Daily Living | AMCI  AD | AUC  sensitivity  specificity |
| 6 | Kim et al. | 2019 | South Korea | case control study | Diagnosis | AD: 35 (9/26)  Other neurological disorders: 18 (6/12)  cognitively unimpaired: 26 (4/22) | AD: 75.8±9.9  Other neurological disorders: 76.9±7.5  cognitively unimpaired: 68.9±5.7 | (1) Aβ in nasal secretions | (1) CDR  (2) MMSE  (3) Aβ deposition on PET  (4) APOE ε4 carrier | AD | AUC  sensitivity  specificity |
| 7 | Velayudhan et al. | 2015 | UK | case control study | Diagnosis | AD: 54 (30/24)  non-demented controls: 40 (18/22) | AD: 73.5±11  non-demented controls: 70.5±9 | (1) UPSIT(University of Pennsylvania Smell Identification Test)  (2) B-SIT  (3) 3 item Pocket-Smell test | (1) MMSE  (2) BADL (Bristol Activities of daily living)  (3) NPI (neuropsychiatric Inventory) | AD | AUC  sensitivity  specificity |
| 8 | Mi et al. | 2023 | China | cross sectional study | Diagnosis | nonMCI (naMCI): 136 (81/55)  MCI: 188 (100/88)  AD: 42 (27/15) | nonMCI (naMCI): 60.43±7.61  MCI: 62.45±7.15  AD: 64.69±9.17 | (1) Chinese Smell Identification Test (CSIT) | (1) MMSE  (2) MoCA (Montreal Cognitive Assessment)  (3) CDR scales  (4) Activity of Daily Living scale | MCI  AD | AUC  OR  sensitivity  specificity |
| 9 | Suzuki et al. | 2004 | Japan | case control study | Diagnosis | AD: 85 (NA)  Non-demented: 30 (NA) | AD: 76.3±7.2  Non-demented: 74.8±8.5 | (1) CC-SIT (Cross-Cultural Smell Identification test)  (2) P-SIT (picture-based smell identification test) | (1) MMSE | AD | AUC  sensitivity  specificity |
| 10 | Kjelvik et al. | 2007 | Norway | case control study | Diagnosis | AD (all): 39 (14/25)  AD (MMSE>=24): 17 (8/9)  Controls: 52 (15/37) | AD (all): 75 [54-89]  AD (MMSE>=24): 73 [54-86]  Controls: 78 [55-91] | (1) B-SIT | (1) MMSE  (2) blood test  (3)cerebral CT or MRI | AD | sensitivity  specificity  AUC |
| 11 | JIMBO et al. | 2011 | Japan | cross sectional study | Diagnosis | AD: 100 (31/69)  Control: 17 (6/11) | AD: 79.29±5.78  Control: 75.86±7.97 | (1) Odor Stick Identification Test for  the Japanese (OSIT-J) | (1) ADAS-cog  (2) Cerebrospinal fluid (CSF) collection  (3) Single-photon emission computed  tomography (SPECT) | AD | OR  95%CI  AUC  sensitivity  specificity |
| 12 | Makowska et al. | 2011 | Poland | case control study | Diagnosis | Young controls: 30 (15/15)  Elderly controls: 30 (15/15)  AD: 30 (15/15) | Young controls: 26.20±4.97  Elderly controls: 72.33±6.29  AD: 72.27±6.09 | (1) Pocket Smell Test (PST) | (1) ADAS-cog  (2) MMSE | AD | sensitivity  specificity |
| 13 | Fukumoto et al. | 2022 | Japan | cohort study | Diagnosis | Healthy controls: 100 (33/67)  MCI group: 61 (18/43)  AD group: 62 (19/43) | Healthy controls: 57.4±11.7  MCI group: 72.8±10.6  AD group: 76.3±8.8 | (1) DESK tool | (1) MoCA-J  (2) MMSE | MCI  AD | sensitivity  specificity  AUC |
| 14 | Eibenstein et al. | 2005 | Italy | case control study | Diagnosis | Normal subjects: 29 (11/18)  aMCI: 29 (10/19) | Normal subjects: 68.8±5.4  aMCI: 71.6±5.3 | (1) SSST | (1) MMSE  (2) CDR  (3) GDS (Geriatric Depression Scale) | MCI | ROC  sensitivity  specificity |
| 15 | Churnin et al. | 2019 | America | cross sectional study | Diagnosis | Normal: 1064 (489/575)  Hyposmia: 245 (136/109)  Anosmia: 67 (50/17)  Olfactory Dysfunction: 312 (26/286) | Normal: 68.7±6.4  Hyposmia: 71.6±7.1  Anosmia: 75±6.2  Olfactory Dysfunction: 72.3±7.1 | (1) Pocket Smell Test (PST) | (1) AFT  (2) DSST  (3) CERAD | MCI  Dementia | OR  95%CI |
| 16 | Quarmley et al. | 2017 | America | case control study | Diagnosis | AD: 262 (98/164)  MCI: 174 (82/92)  Healthy control: 292 (89/203) | AD: 75.18±8.22  MCI: 72.46±8.57  Healthy control: 70.96±8.74 | (1) SS-OIT (Sniffin' Sticks Odor Identification Test) | (1) MoCA  (2) MMSE | AD  MCI | sensitivity  specificity  AUC |
| 17 | Pusswald et al. | 2022 | Austria | retrospective longitudinal study  case control study | Prognosis | Healthy control: 16 (5/11)  Subjective cognitive decline: 23 (13/10)  naMCI: 75 (32/43)  aMCI: 59 (27/32)  2-year follow-up：59 patients with aMCI progressed.  AD: 18 (NA)  non-AD: 41 (NA) | Healthy control: 56.3±5.8  Subjective cognitive decline: 66.1±10.0  naMCI: 67.9±8.2  aMCI: 68.9±7.4 | (1) ASOF (SOC+SRP+ORQ)  (2) SS-OIT | (1) Wortschatztest(WST)  (2) BDI-II  (3) MMSE  (4) NTBV  (5) MBNT  (6) VSRT  (7) TMT-A  (8) TMT-B | aMCI  naMCI | AUC  OR  95%CI |
| 18 | Pellkofer et al. | 2018 | Germany | case control study | Diagnosis | AD: 12 (NA)  MCI: 13 (NA)  cognitively normal controls(CN): 10 (NA) | AD: 67-85  MCI: 42-86  cognitively normal controls(CN): 55-71 | (1) Sniffin Sticks test  (2) Biopsies of nasal mucosa  (3) Immunohistochemistry and BSC4090 labeling | (1) CSF amyloid-β 42 total tau,  and p-tau 181  (2) MMSE  (3) brain MRI or cranial computer tomography imaging | AD  MCI | AUC  sensitivity  specificity |
| 19 | Moon et al. | 2016 | South Korea | case control study | Diagnosis | CDR: clinical dementia rating  CDR 0(cognitively healthy and did not exhibit depression): 9 (4/5)  CDR 0.5(MCI): 13 (4/9)  CDR 1: 11 (6/5)  Depression: 8 (2/6) | CDR: clinical dementia rating  CDR 0: 63 [50-74]  CDR 0.5(MCI): 68 [56-79]  CDR 1: 69 [57-80]  Depression: 61.5 [56-66] | (1) intranasal biopsy | (1) CDR  (2) BDI-II  (3) MMSE-Korea  (4) ADAS-Cog-Korea | MCI | AUC  sensitivity  specificity |
| 20 | Liang et al. | 2016 | China | cross sectional study | Diagnosis | All: 1782 (818/964)  Cognitive Normal: 1437 (659/778)  MCI: 345 (159/186) | All: 70.1±7.1  Cognitive Normal: 69.4±6.8  MCI: 73.0±7.8 | (1) olfactory identification | (1) CDR  (2) Zung Self-Rating Anxiety Scale  (3) Activity of Daily Living (ADL) scale  (4) CSED  (5) MMSE  (6) Go/No Go Task  (7) Stick Test  (8) Modified Common Objects Sorting Test  (9) AVLT  (10) Modified Fuld Object Memory Evaluation  (11) Trail-making test A&B  (12) RMB (Chinese currency) test | MCI | OR  95%CI |
| 21 | Fusetti et al. | 2010 | Italy | Cohort prospective study | Diagnosis  Prognosis | T0: First assessment  aMCI: 29 (10/19)  T1:18-month follow-up  aMCI: 20 (NA)  aMCI→AD: 9 (NA) | T0:First assessment  aMCI: 70.8±5.7  T1: 18-month follow-up  NA | (1) Sniffin‘Sniffing Screen Test，SSST  (2) Sniffin’Sniffin‘Screen Test，SSET | (1) MMSE  (2) MDB | aMCI  AD | ROC  sensitivity  specificity |
| 22 | Djordjevic et al. | 2008 | Canada | case control study | Diagnosis | NEC (normal elderly control): 33 (17/16)  MCI: 51 (25/26)  AD: 27 (14/13) | NEC (normal elderly control): 73.7 [63-87]  MCI: 75.4 [59-86]  AD: 77.0 [55-88] | (1) detection threshold  (2) odor discrimination  (3) odor identification  (4) modified version of a discrimination test  (5) UPSIT | (1) MMSE  (2) digit and spatial span tests  (3) Similarities subtest from the Wechsler Adult Intelligence Scale-III  (4) Block Design subtest from the Wechsler Adult Intelligence Scale-III  (5) RAVLT  (6) Visual Reproductions subtest from the Wechsler Memory Scale-III  (7) Boston Naming Test  (8) FAS and animal naming  (9) Geriatric Depression Scale (GDS) | MCI  AD | sensitivity  specificity |
| 23 | Conti et al. | 2013 | Italy | cohort study | Prognosis | Baseline：  MCI olfactory impaired: 53 (25/28)  MCI olfactory normal: 35 (14/21)  Control: 46 (20/26)  2-year follow-up：progression to AD  Non-progression: 57 (NA)  Progression: 27 (NA) | Baseline：  MCI olfactory impaired: 75.9±6.1  MCI olfactory normal: 71.1±6.6  Control: 73.7±7.3  2-year follow-up：  Non-progression: 72.5±6.8  progression: 76.5±5.8 | (1) CA-SIT Smell Identification Test | (1) MMSE  (2) CDR  (3) ADAS-Cog-Italian version  (4) the IADL and BADL scales  (5) Geriatric Depression Scale  (6) Neuropsychiatric Inventory  (7) Novelli’s short story—learning and recall  (8) Raven’s colored matrices  (9) Trail Making Test A and B  (10) Rey figure copy and recall  (11) semantic and phonemic fluency  (12) Clock drawing test | AD | AUC  sensitivity  specificity |
| 24 | Devanand et al. | 2000 | America | cohort study | Prognosis | Baseline：  MCI: 90 (43/47)  normal comparison subjects: 45 (23/24)  Follow-up：20-month an average (SD=12)  18 of 40 patients with MCI progressed to AD. | Baseline：  MCI: 66.7±10.7  normal comparison subjects: 64.0±10.0 | (1) University of Pennsylvania Smell Identification Test | (1) WAIS-R  (2) Wechsler Memory Scale  (3) Selective Reminding Test  (4) Rosen Drawing Test  (5) Controlled Oral Word Association Test  (6) category naming test from the Boston Diagnostic Aphasia Evaluation  (7) Boston Naming Test  (8) BVRT  (9) Target Finding Test  (10) shape and letter cancellation tasks | MCI  AD | sensitivity  specificity |
| 25 | Dong et al. | 2022 | China | cross-sectional study | Diagnosis | All: 4481 (NA)  dementia: 139 (NA)  AD: 91 (NA) | All: 71.1±4.9  non-Dementia: 71.0±4.8  Dementia: 74.4±6.7 | (1) SSIT (16-item Sniffin' Sticks Identification Test) | (1) AD8 interview (the Ascertain Dementia 8-item questionnaire) | all-cause Dementia  AD | AUC  95%CI  sensitivity  specificity |

## Supplementary Figures

**
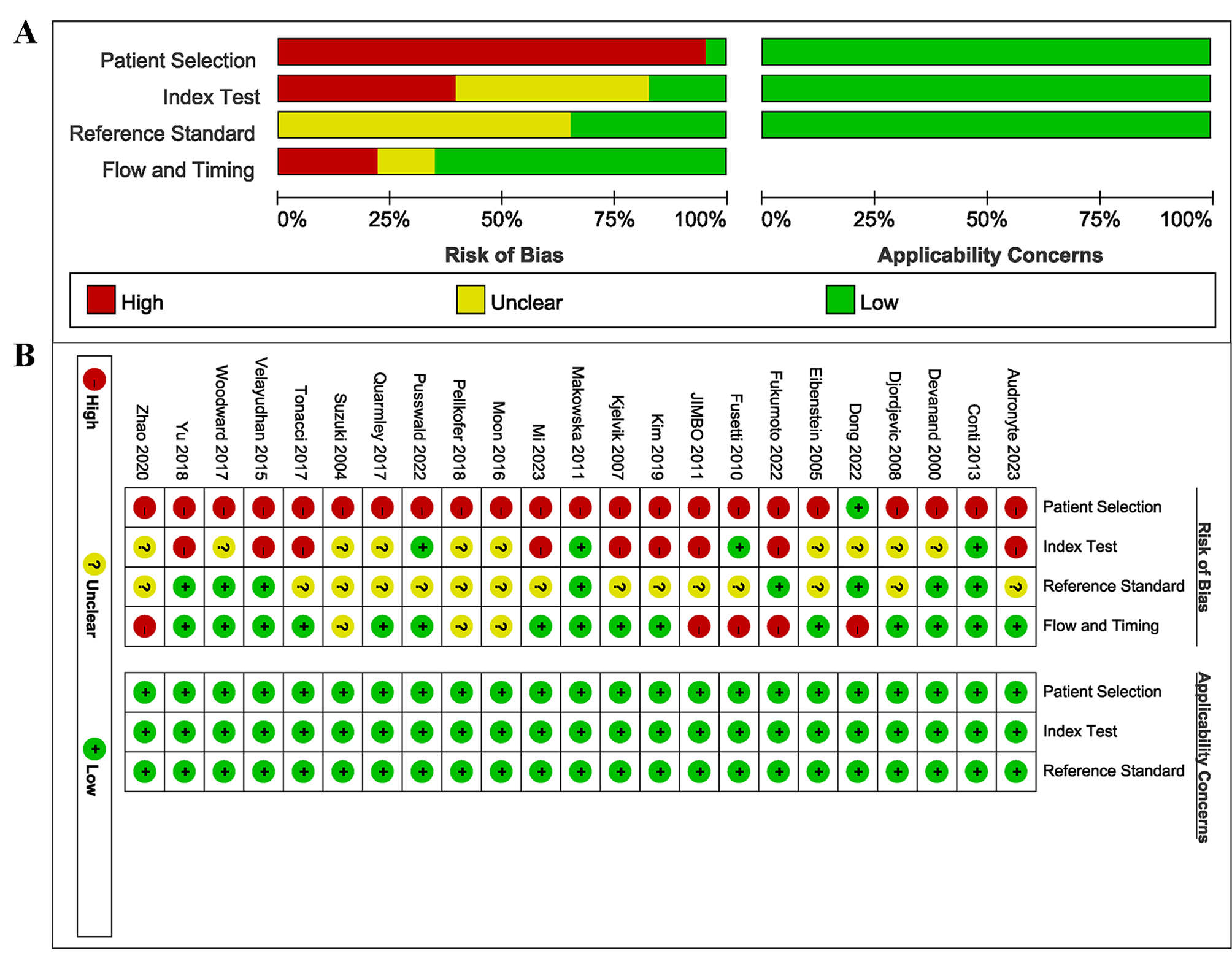
**

**Figure S1** The QUADAS-2 standard used for quality assessment as shown


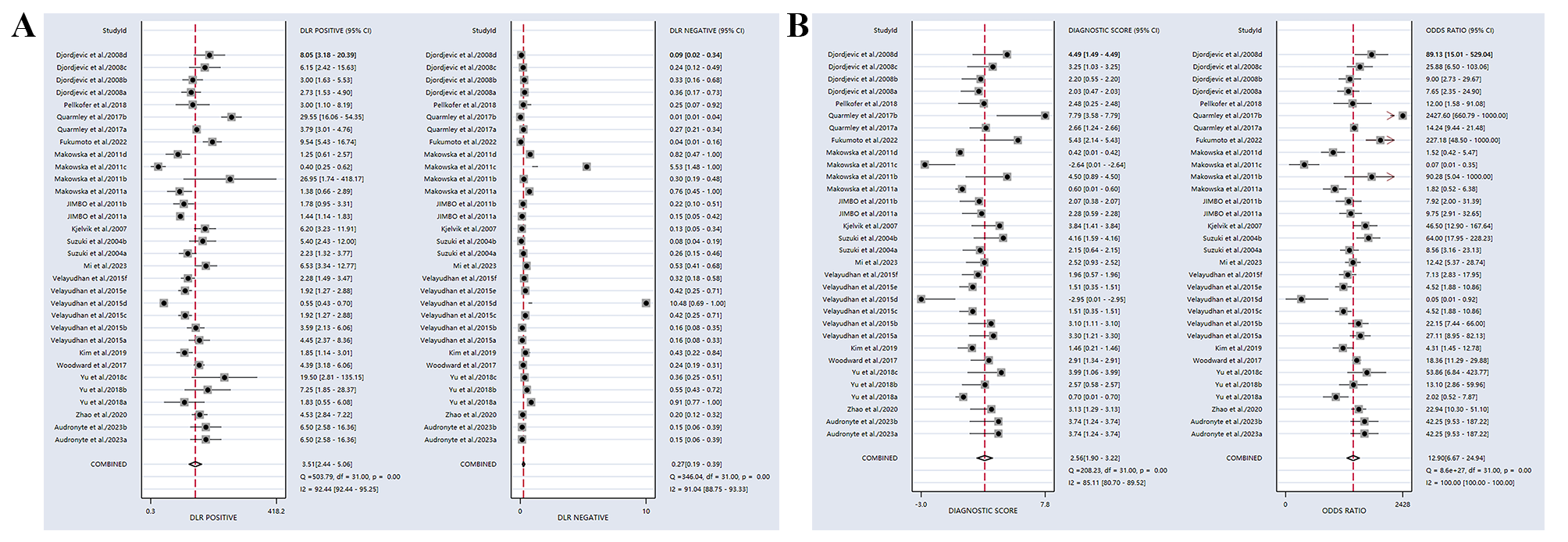


**Figure S2** (A) The pooled PLR and NLR for diagnosing AD using olfactory function testing**;** (B)The combined DOR for diagnosing AD using olfactory function testing


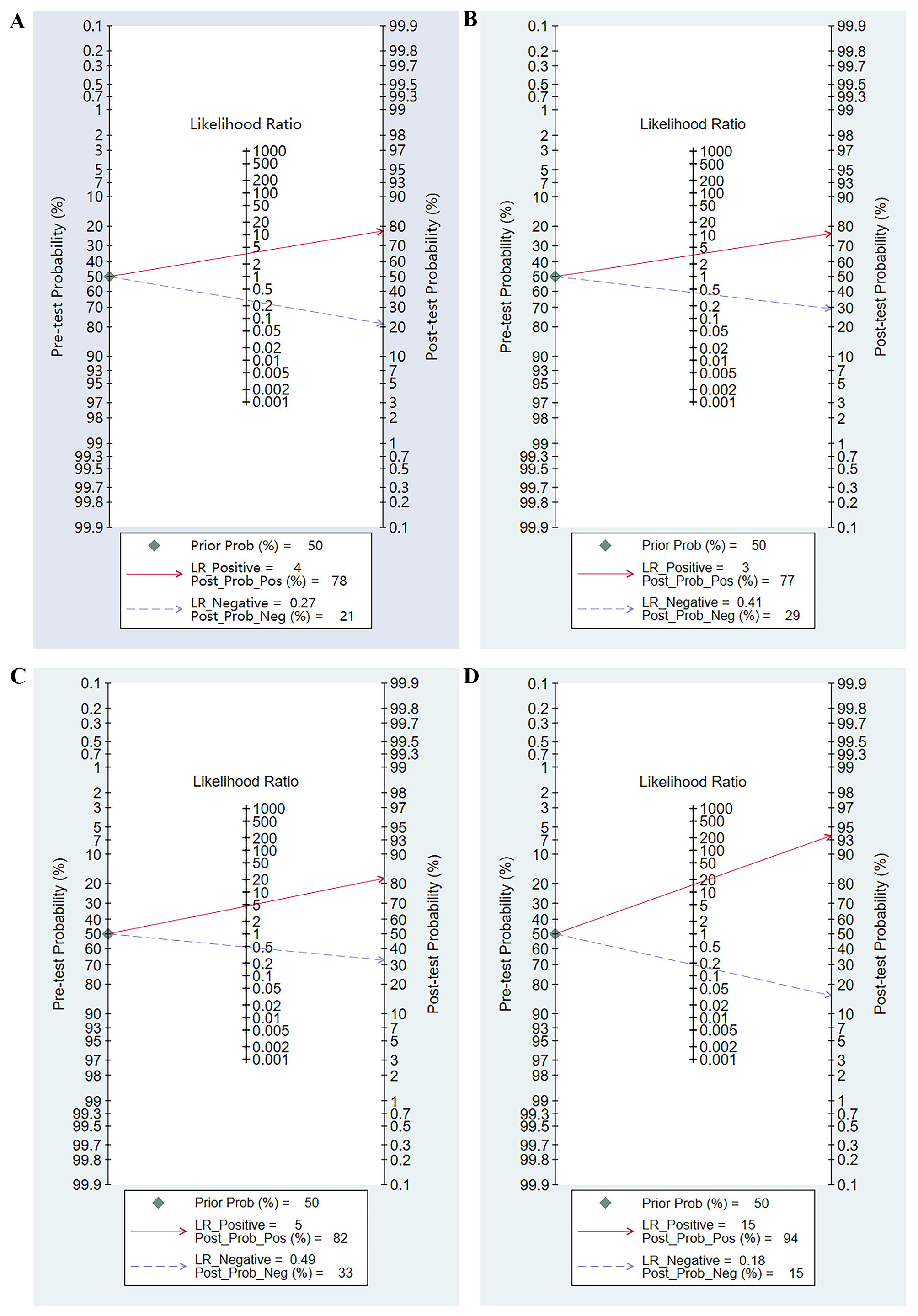


**Figure S3 (A)** Clinical Effect for diagnosing AD using olfactory function testing; **(B)** Clinical Effect of MCI diagnosed by olfactory function testing; (C) Clinical Effect of olfactory function testing for the diagnosis of AD compared to MCI; (D) Clinical Effect of olfactory function testing combined with other indicators for diagnosing AD or MCI


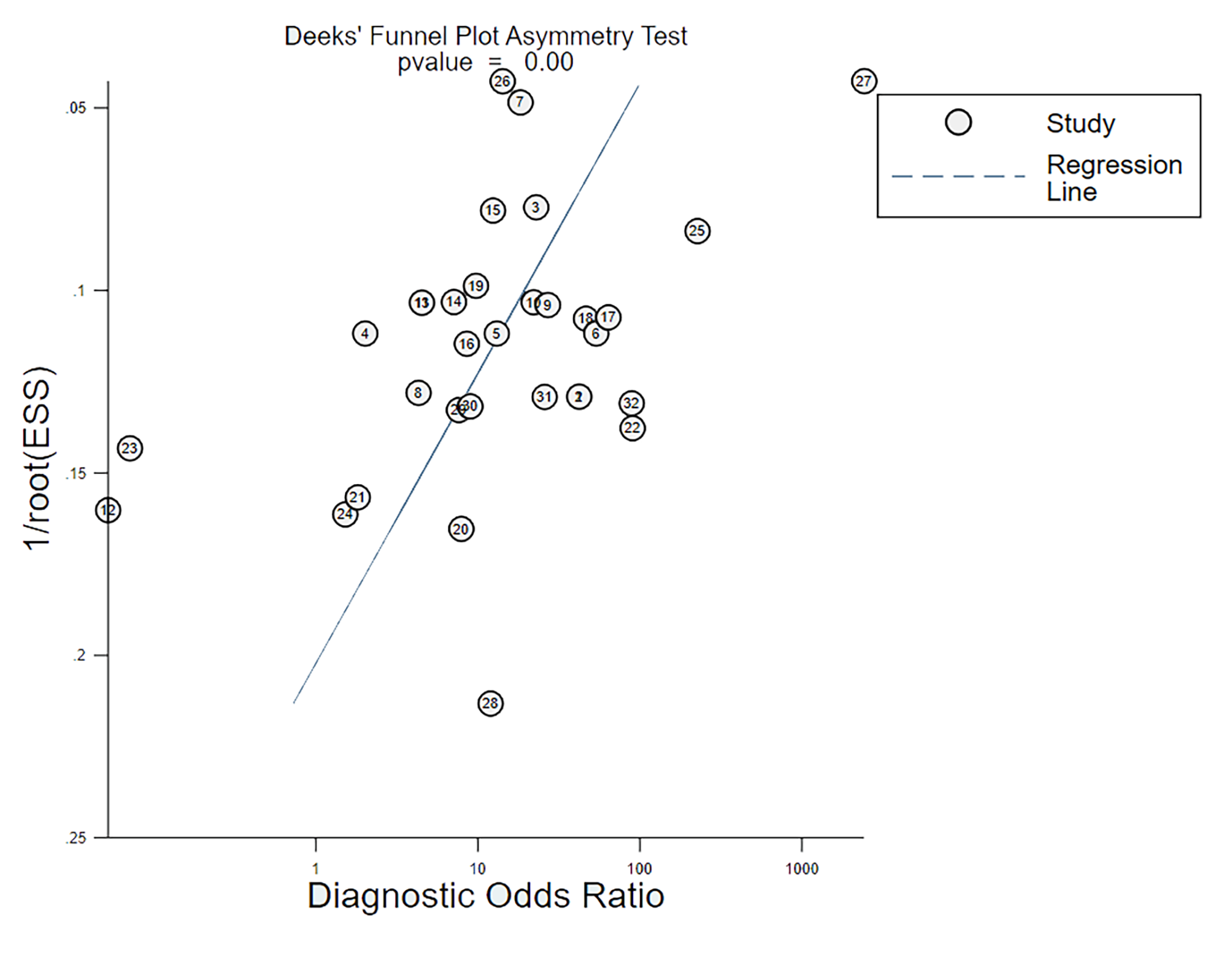


**Figure S4** Publication Bias of Efficiency Analysis of Olfactory Function Testing for Diagnosing Healthy People and AD


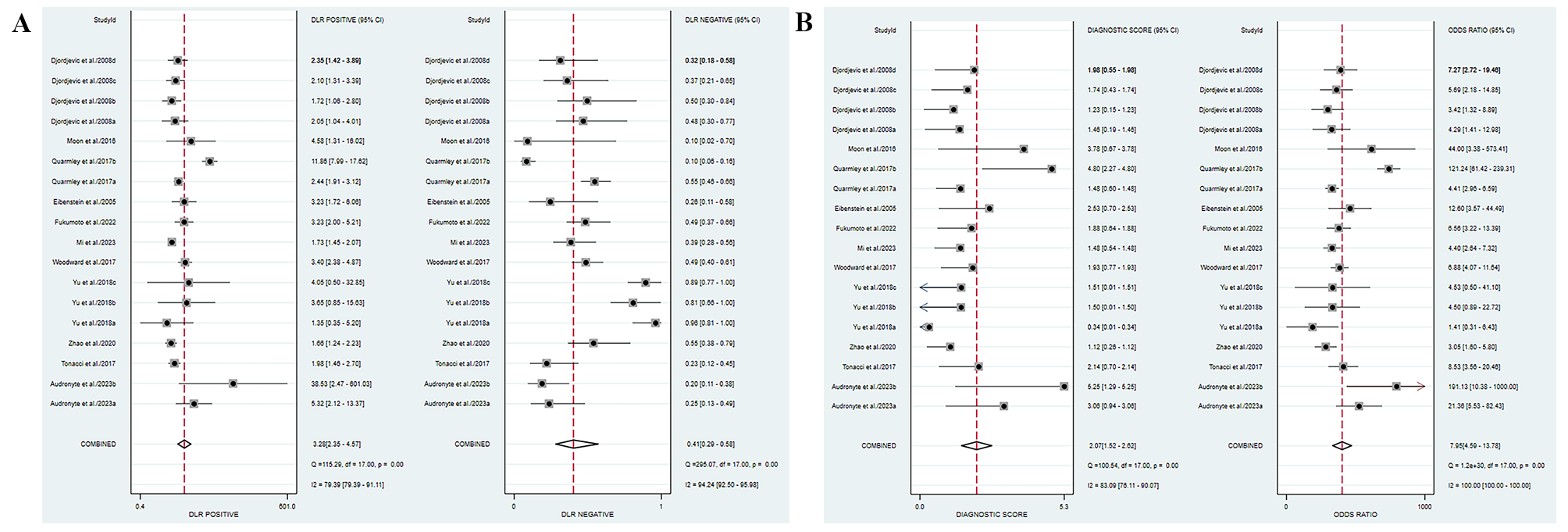


**Figure S5** (A) The pooled PLR and NLR of MCI diagnosed by olfactory function testing; (B) The combined DOR of MCI diagnosed by olfactory function testing


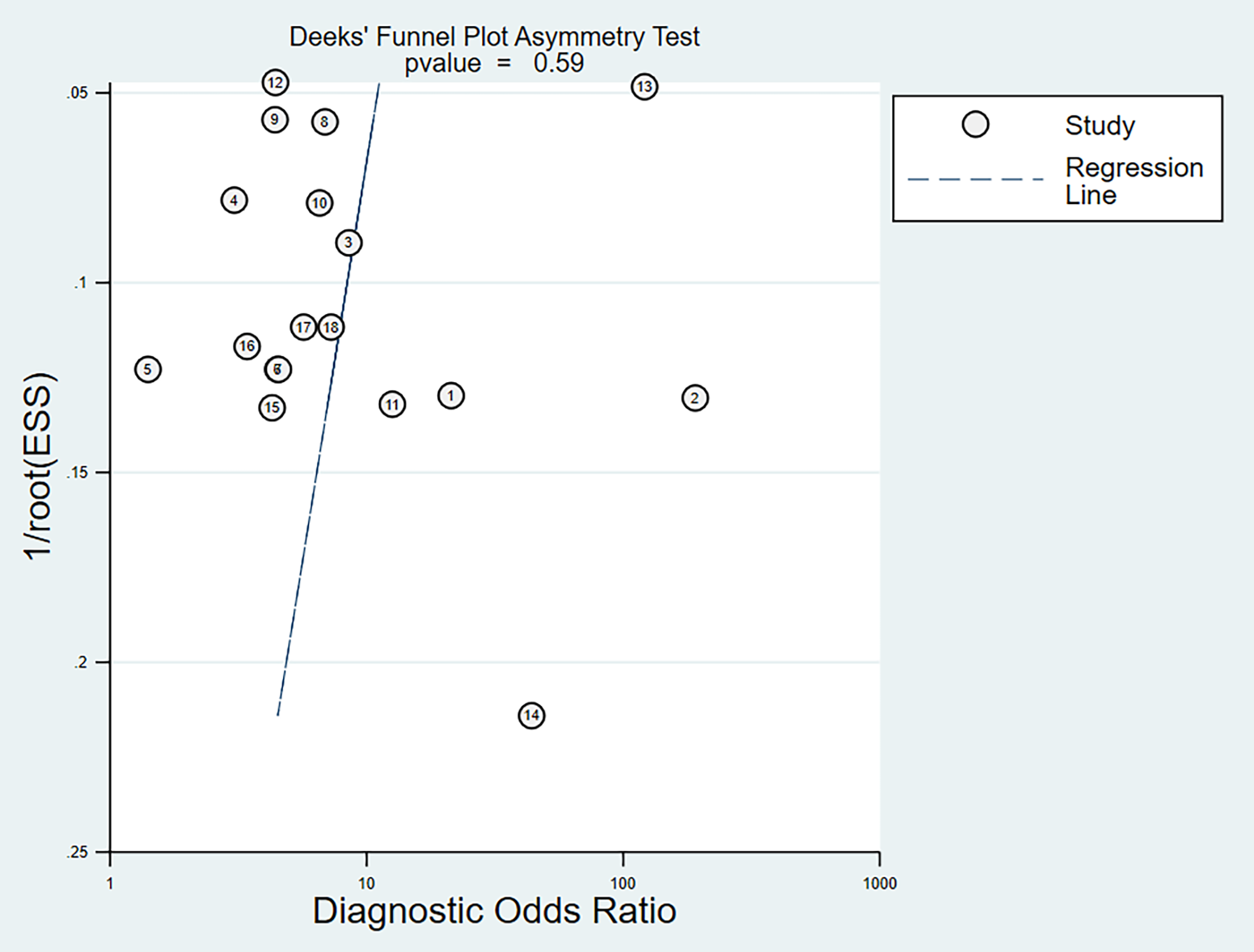


**Figure S6** Publication Bias of Efficiency Analysis of Olfactory Function Testing for Diagnosing Healthy People and MCI


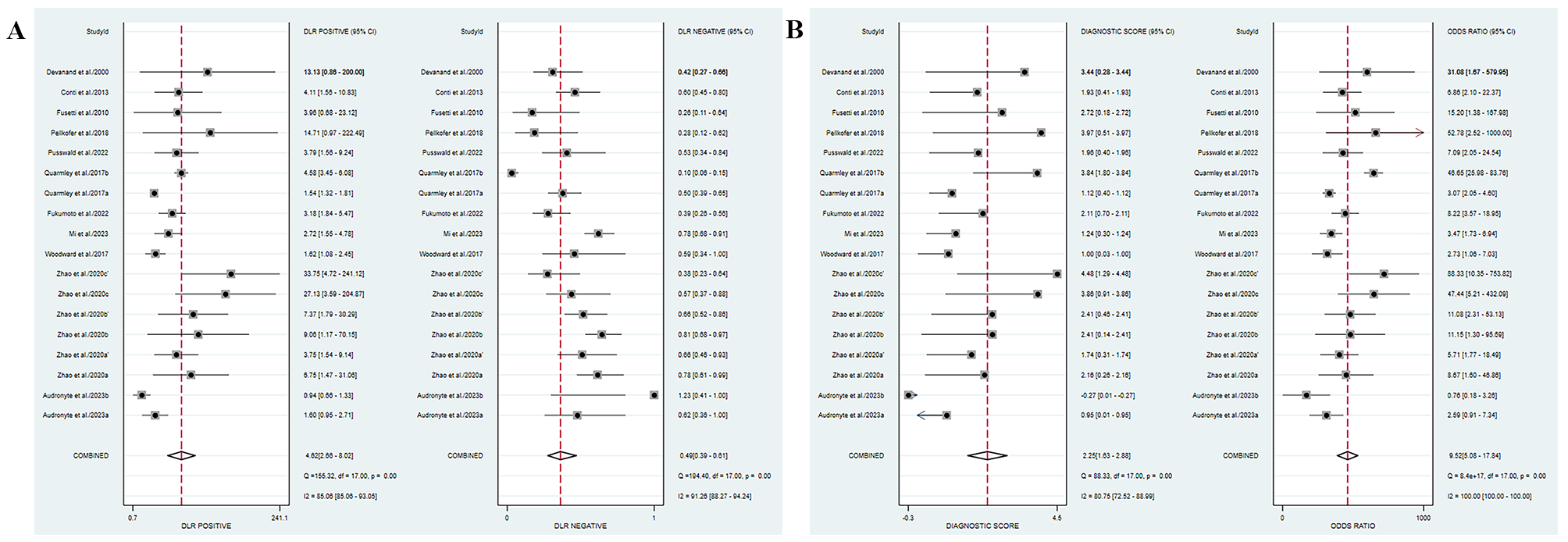


**Figure S7 (A)** The pooled PLR and NLR of olfactory function testing for the diagnosis of AD compared to MCI**; (B)**The combined DOR of olfactory function testing for the diagnosis of AD compared to MCI


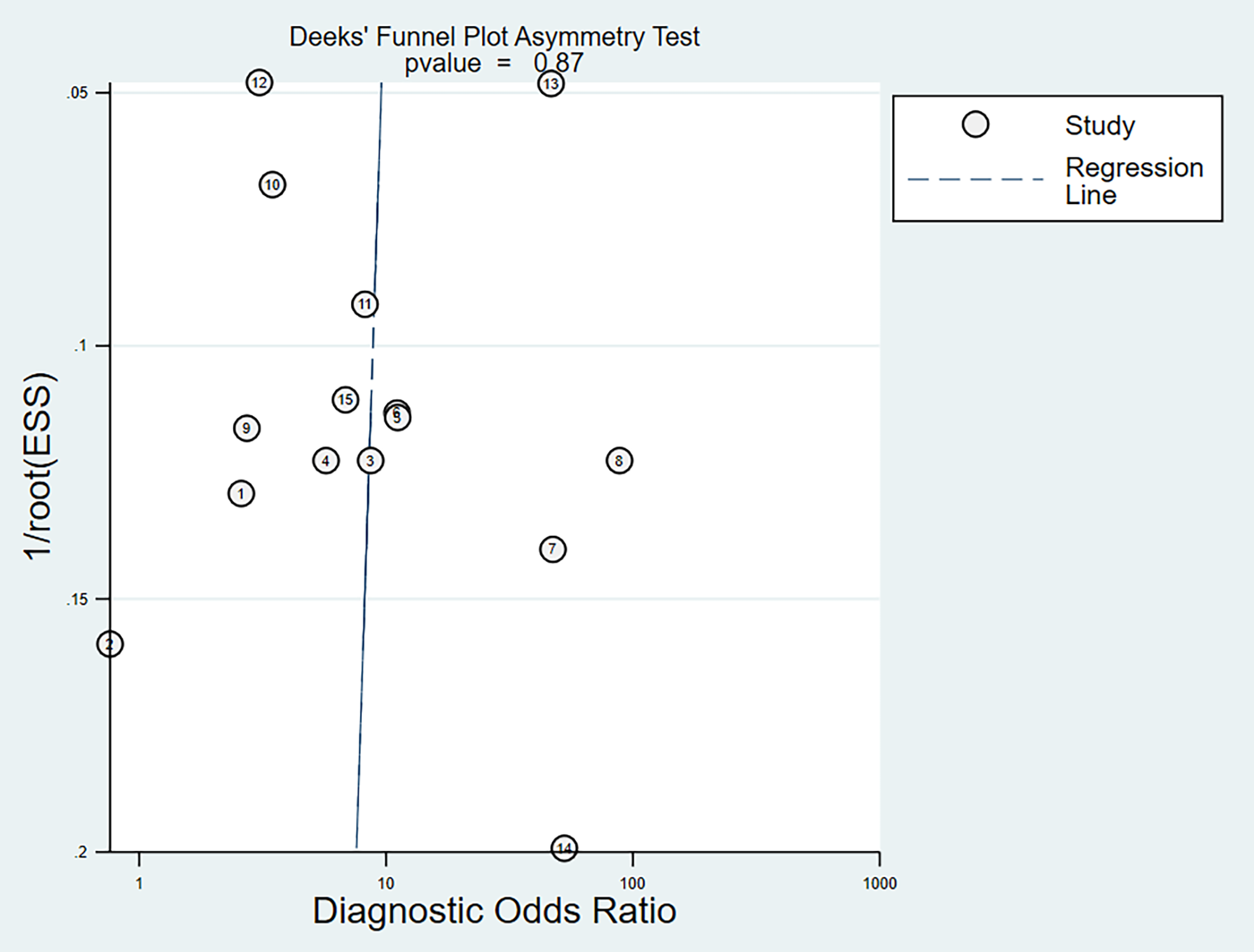


**Figure S8** Publication Bias of Efficiency Analysis of Olfactory Function Testing for Diagnosing MCI and AD


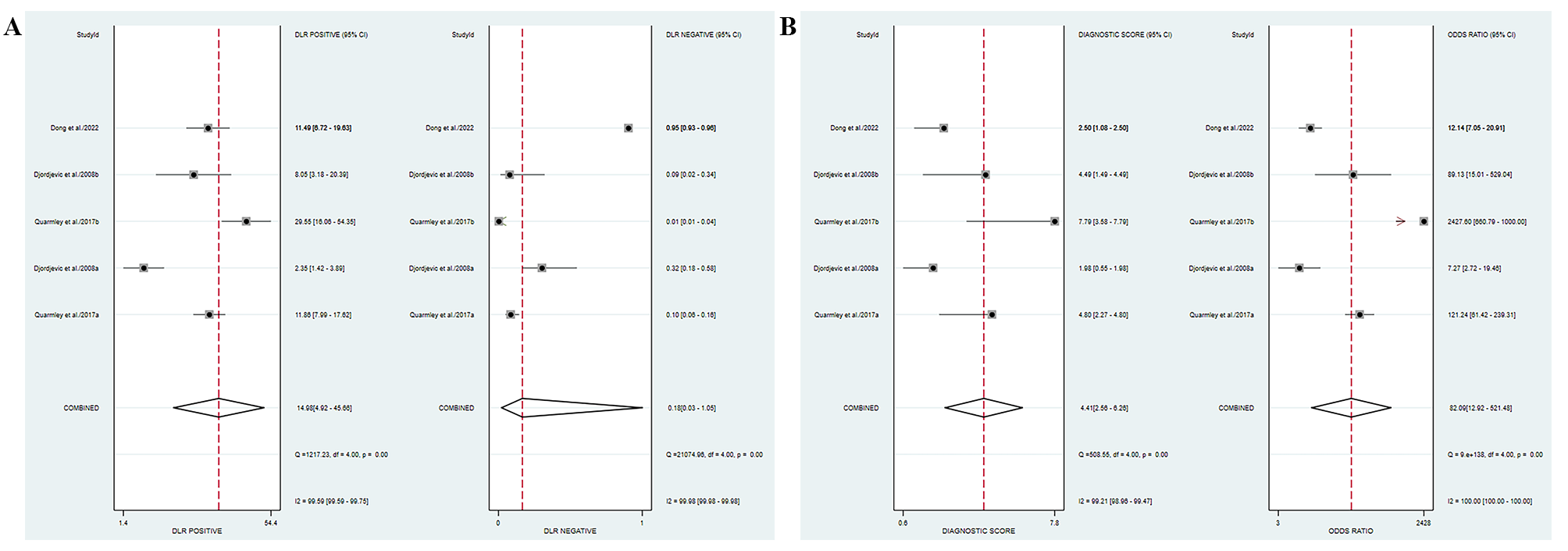


**Figure S9 (A)** The pooled PLR and NLR of olfactory function testing combined with other indicators for diagnosing AD or MCI**; (B)** The combined DOR of olfactory function testing combined with other indicators for diagnosing AD or MCI


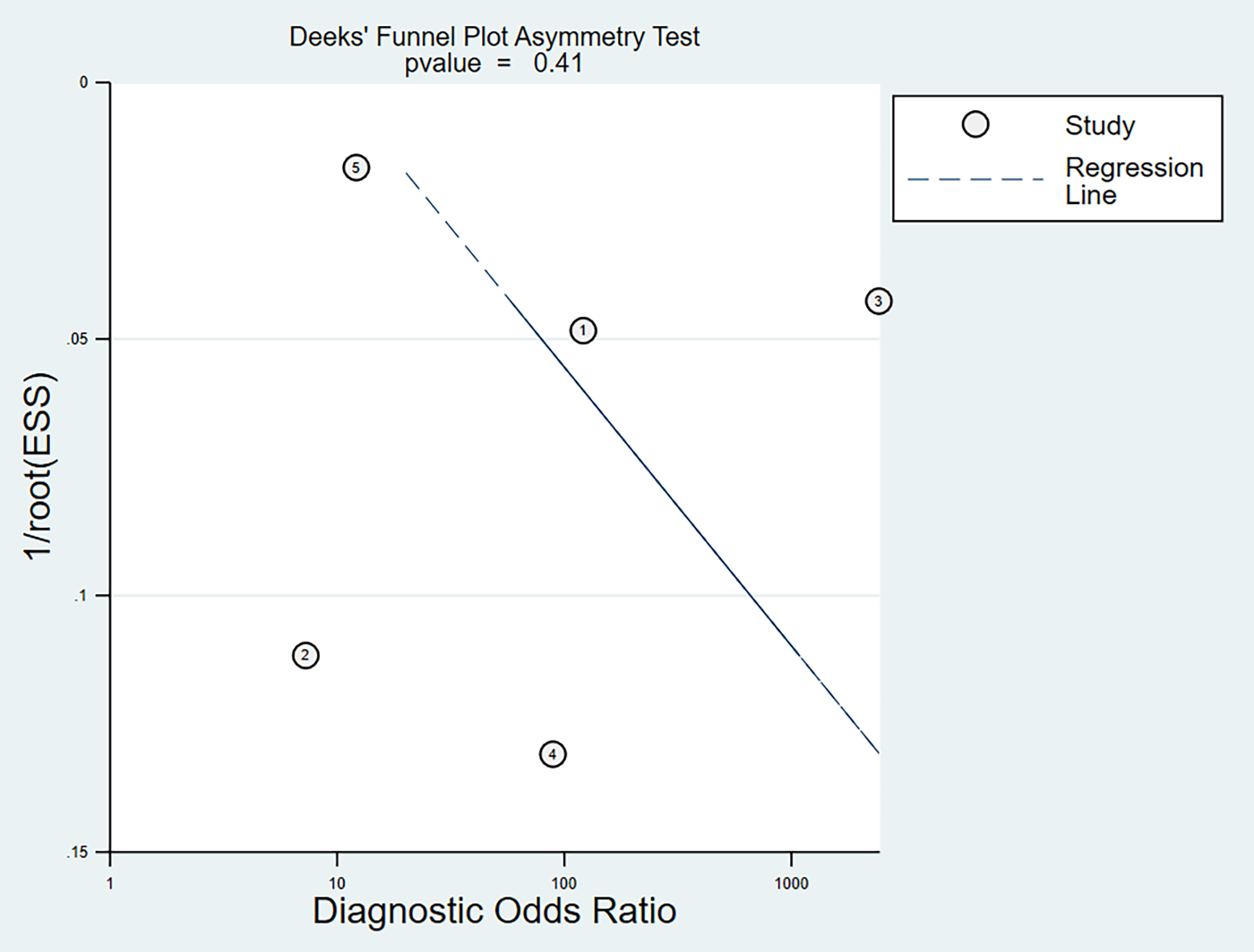


**Figure S10** Publication Bias of Efficiency Analysis of Olfactory Function Testing Combined with other Tests for Diagnosing AD or MCI


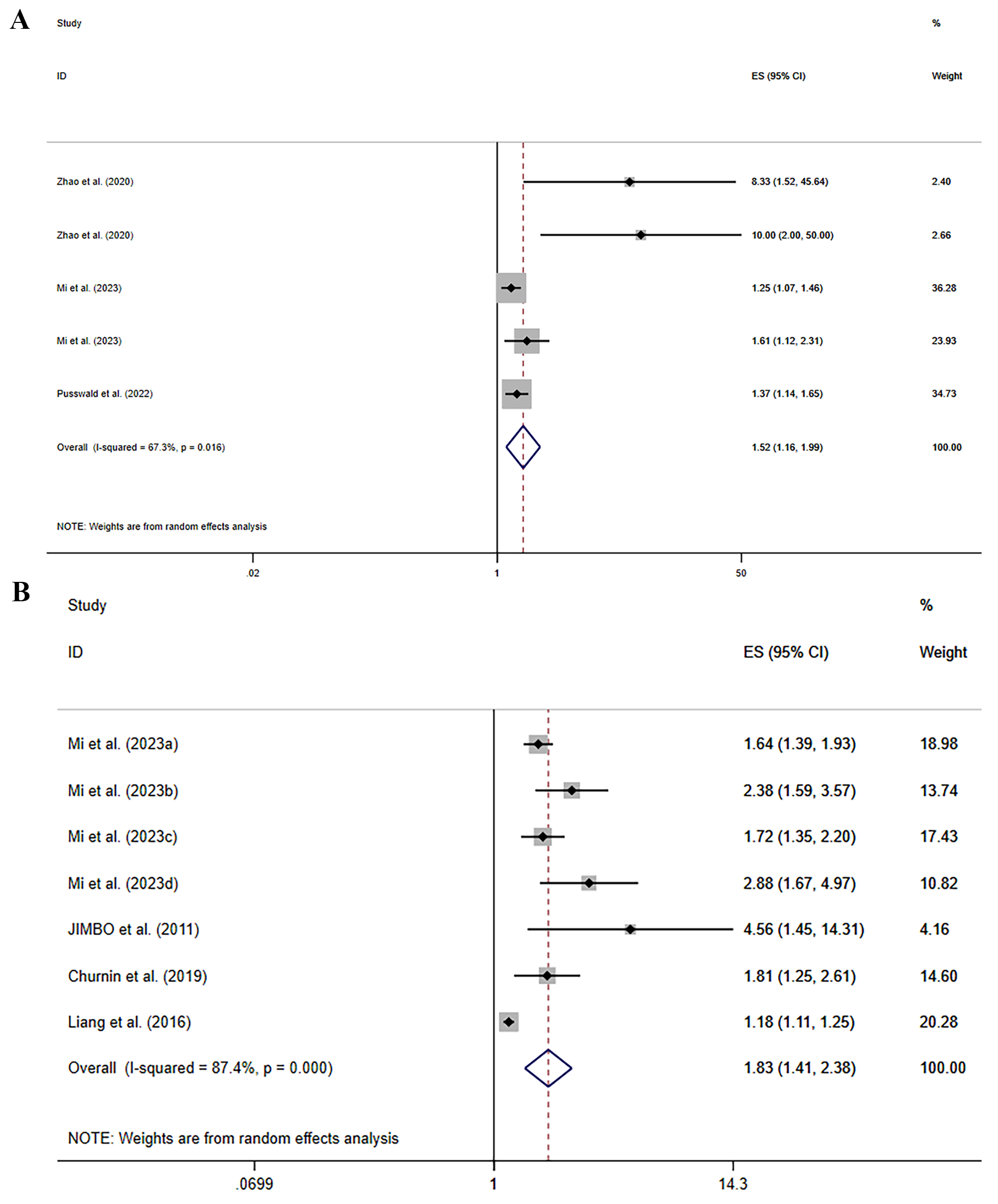


**Figure S11 (A)** Relationship Between Olfactory Function and Progression to MCI and AD in Normal Individuals**; (B)** Relationship Between Olfactory Function and Progression of MCI to AD Patients
